# Positive selection and ancient duplications in the evolution of class B floral homeotic genes of orchids and grasses

**DOI:** 10.1186/1471-2148-9-81

**Published:** 2009-04-21

**Authors:** Mariana Mondragón-Palomino, Luisa Hiese, Andrea Härter, Marcus A Koch, Günter Theißen

**Affiliations:** 1 Department of Genetics, Friedrich Schiller University Jena, Philosophenweg 12, D-07743 Jena, Germany; 2 Institute for Plant Science, Ruprecht Karls University, Im Neuenheimer Feld 360, 69120 Heidelberg, Germany

## Abstract

**Background:**

Positive selection is recognized as the prevalence of nonsynonymous over synonymous substitutions in a gene. Models of the functional evolution of duplicated genes consider neofunctionalization as key to the retention of paralogues. For instance, duplicate transcription factors are specifically retained in plant and animal genomes and both positive selection and transcriptional divergence appear to have played a role in their diversification. However, the relative impact of these two factors has not been systematically evaluated. Class B MADS-box genes, comprising *DEF*-like and *GLO*-like genes, encode developmental transcription factors essential for establishment of perianth and male organ identity in the flowers of angiosperms. Here, we contrast the role of positive selection and the known divergence in expression patterns of genes encoding class B-like MADS-box transcription factors from monocots, with emphasis on the family Orchidaceae and the order Poales. Although in the monocots these two groups are highly diverse and have a strongly canalized floral morphology, there is no information on the role of positive selection in the evolution of their distinctive flower morphologies. Published research shows that in Poales, class B-like genes are expressed in stamens and in lodicules, the perianth organs whose identity might also be specified by class B-like genes, like the identity of the inner tepals of their lily-like relatives. In orchids, however, the number and pattern of expression of class B-like genes have greatly diverged.

**Results:**

The *DEF*-like genes from Orchidaceae form four well-supported, ancient clades of orthologues. In contrast, orchid *GLO*-like genes form a single clade of ancient orthologues and recent paralogues. *DEF*-like genes from orchid clade 2 (*OMADS3*-like genes) are under less stringent purifying selection than the other orchid *DEF*-like and *GLO*-like genes. In comparison with orchids, purifying selection was less stringent in *DEF*-like and *GLO*-like genes from Poales. Most importantly, positive selection took place before the major organ reduction and losses in the floral axis that eventually yielded the zygomorphic grass floret.

**Conclusion:**

In *DEF*-like genes of Poales, positive selection on the region mediating interactions with other proteins or DNA could have triggered the evolution of the regulatory mechanisms behind the development of grass-specific reproductive structures. Orchidaceae show a different trend, where gene duplication and transcriptional divergence appear to have played a major role in the canalization and modularization of perianth development.

## Background

One important goal of contemporary biology is to understand how changes in developmental processes generate evolutionary novelties at the morphological level. The growing field of evolutionary developmental biology ('evo-devo'), approaches this question by determining how changes in the number, sequence and expression of developmental regulatory genes bring about formation of new structures. In plants and animals, these developmental regulatory factors have expanded during evolution (e.g. by gene and genome duplication) to form large and diverse gene families linked by complex genetic and physical interactions [1-3].

Mutations in transcriptional regulators of development often do not significantly affect the complete organism because their function is generally confined to a single category of organs or modules [4]. Thus, it has been hypothesized that developmental transcription factors are more likely to evolve new functions and so coordinate the development of viable morphological novelty [4]. The importance of duplication and diversification of genes encoding transcription factors (e.g. Hox genes) is substantiated by genomic analyses showing that these kinds of genes are specifically retained in plant [5] and animal genomes [6,7]. Additionally, these genes show diverging patterns of expression, unequal rates of substitution and positive selection [6-11].

Positive selection is also involved in the diversification of several groups of plant developmental transcription factors [12-17]. Recent research has focused on those encoded by members of the MIKC-type MADS-box gene family because of their key role in the development and evolutionary diversification of the angiosperm flower [18-22]. Thus, characterizing their patterns of molecular evolution is essential to understanding their function and the mechanisms of morphological evolution. Because different functional classes of MADS-box genes form distinct clades [23-25], their phylogeny is an important aid to identify and test hypotheses explaining the different selective regimes that are generally considered to drive their evolution.

The plant-specific proteins encoded by MIKC-type MADS-box genes have an unique and highly-conserved domain structure that includes MADS- (M-), intervening (I-), keratin-like (K-) and C-terminal (C-) domains [26]. The MADS-domain is mostly involved in DNA-binding and, together with the I-domain, mediates the formation of dimers. The K-domain plays an important role in protein – protein interaction during both dimerization and the formation of multimeric complexes. The C-terminal domain is the most variable region. In some cases it is involved in transcription activation, but it may also be involved in multimeric complex formation (reviewed in [26]).

The ABCDE model of flower development (reviewed in [26]), describes the genetic interactions of the five major classes of floral homeotic selector genes termed class A, B, C, D and E genes, almost all of which are MIKC-type genes. Each of these gene classes determines the identity of different floral organs: Class A and E genes specify sepals; class A, B and E genes determine petals; the combination of class B, C and E genes specifies stamens (male reproductive organs); class C and E genes determine carpels (female reproductive organs); and class D genes determine ovules.

Of special interest to our study are class B MADS-box genes encoding transcription factors, key to the specification of petal and stamen identity [27-32]. A gene duplication event that preceded the origin of extant angiosperms gave rise to *DEF*- and *GLO*-like genes, the two major lineages of class B genes in angiosperms [33-35]. The regulatory role of class B genes in some model plants such as *Arabidopsis thaliana *and *Antirrhinum majus *involves obligatory heterodimerization of proteins from the DEF and GLO lineages [27,29,36]. Moreover, these heterodimers form higher order complexes with other classes of MADS-domain proteins [37-39].

Recent analyses of the molecular evolution of class B-like MADS-box genes in angiosperms detected positive selection after two key duplication events that generated first *DEF*- and *GLO*-like genes and later eu*AP3*-type and *TM6*-type genes, which are the major sublineages of *DEF*-like genes [22]. The analysis of Hernández-Hernández et al. showed that during evolution, positive selection probably modified the central property of protein complex formation because most of the selected sites belong to the K-domain mediating protein – protein interactions in the complexes of MADS-domain transcription factors [22]. Thus, the evolutionary emergence and divergence of *DEF*- and *GLO*-like genes after duplication enabled the formation of obligate heterodimeric complexes involved in the determination of floral organ identity, while the evolution of the class B gene lineages of eu*AP3*-type and *TM6*-type genes may be associated with the morphological canalization of the core eudicot flower [22,40].

Flowers of many monocots are actinomorphic, with two trimerous whorls of highly similar petaloid organs called tepals. In contrast, at least three kinds of organ identity exist in the zygomorphic orchid perianth: in the first floral whorl there are three outer tepals (T1–T3; often also termed 'sepals'). In the second whorl there are two lateral inner tepals (t1, t2; 'petals') and a median inner tepal (t3) called lip or labellum [41,42]. The orchid family is divided into five monophyletic subfamilies that successively diverged from each other: Apostasioideae, Vanilloideae, Cypripedioideae, Orchidoideae and Epidendroideae [43,44]. In each subfamily the three types of perianth organs have specific features, though the lip typically adopts the widest range of morphologies. In *Apostasia*, one of two genera of the Apostasioideae, the lip resembles the other tepals [45], while in flowers of Cypripedioideae the lip is "deeply pouched and inflated" [46].

In contrast, the reproductive organs of the typical grass floret are surrounded (from inner to outer) by two lodicules, palea and lemma following a zygomorphic organization. One or more florets are surrounded by two glumes, thus constituting a spikelet, the characteristic reproductive unit of grass inflorescences [47,48]. Class B gene expression and corresponding homeotic transformations in mutant plants suggest that the lodicules are probably homologous with eudicot petals, even though lodicules are small, often glandular-looking organs that appear very different from typical petals [49,50]. The homologies of palea, lemma and glumes to organs of other angiosperms, such as sepals or prophylls remain controversial [51].

Many monocot species have several copies of *DEF*- and *GLO*-like genes which are expressed differently from their orthologues first characterized in *Antirrhinum majus *and *Arabidopsis thaliana *[35,52]. For example, the development of petaloid tepals in the outer whorl of *Tulipa gesneriana *and other petaloid monocots is probably determined by the heterotopic expression of *DEF- *and *GLO*-like genes [53-56]. Although this research suggests there are alternative ways in which class B proteins regulate the genes encoding them and are associated with novel morphologies, it is still far from clear how MADS-box gene duplication and transcriptional divergence is associated with developmental flexibility and the evolutionary diversification of floral morphology. An opportunity to address this question comes from recent studies in putative class B genes from Orchidaceae (orchids; order Asparagales), which indicate that the high degree of perianth diversity of this family might be associated to duplication of MADS-box genes [57-61]. Specifically, studies on the orchid species *Habenaria radiata, Dendrobium crumenatum, Phalaenopsis equestris *and the hybrid *Oncidium *"Gower Ramsey", have indicated that the petaloid character of the outer tepals of orchid flowers is due to heterotopic expression of class B genes *HrDEF*, *DcOAP3A*, *PeMADS2 *and *PeMADS5*, and *OMADS3*, respectively [57,58,60,61]. In particular, the analyses of *Phalaenopsis equestris *and *Dendrobium crumenatum *indicated that the specific combination of duplicate gene expression in each whorl is associated with development of three distinct groups of organs: the outer and inner lateral tepals and the exceptionally diverse orchid lip [58,60].

Recently, we linked these distinct patterns of expression and organ identity determination with preliminary data on the molecular phylogeny of orchid *DEF*-like genes [42,62]. We argued, that the orchid perianth evolved from a lily-like ancestor as a result of duplication of *DEF*-like genes and subsequent regulatory changes that brought about differential expression of the paralogues. Our model predicts that the extant diversity of the orchid perianth results from changes in expression of some of these four *DEF*-like genes, or from changes in the downstream targets of the proteins that they encode [42]. These two scenarios are not mutually exclusive and the pattern of selection on these genes could help to distinguish between them. We hypothesize that the occurrence of distinct patterns of molecular evolution in each clade of orchid *DEF*-like genes may substantiate the hypothesis that each of them is associated to distinct protein- or DNA-binding capabilities.

The *DEF*- and *GLO*-like genes from the order Poales provide a useful point of comparison for testing this hypothesis because they are essential for specification of stamens and the grass-specific lodicules [49,50] and are expressed in homologous whorls of the grasses and their closest relatives. This indicates that the morphological differentiation of grasses from tepaloid monocots is possibly the result of changes in the downstream targets of class B transcription factors [63].

**Figure 1:**
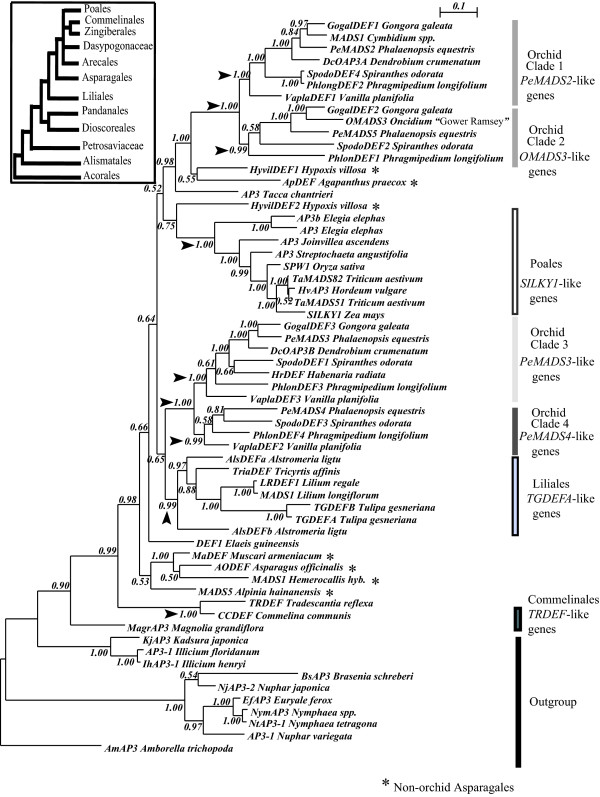
**Phylogeny of monocot *DEF*-like genes based on an alignment of cDNA encoding the MIK-domains**. The figure shows the Bayesian inference phylogeny of those *DEF*-like sequences for which at least 80% of the complete coding sequence is available and the new ones reported here. An appropriate model of nucleotide substitution was selected by Modeltest for the nucleotide sequences encoding the M-, I- and K- domains, and used to infer this phylogeny where the C-terminal domain was excluded. The bars indicate the different clades of *DEF*-like genes from the Orchidaceae, Liliales, Commelinales, Poales and the outgroup. The numbers on every node indicate the Bayesian posterior probabilities (PP). Black arrows emphasize nodes that are discussed in the text. The inset shows a diagram of the monocot relationships based on phylogenetic analyses by [65,66].

With nearly 25,000 and 20,000 species each, Orchidaceae and Poales, respectively constitute two thirds of all monocot species and include some of the most frequently studied groups of plants [47,64]. Molecular phylogenetic analysis have consistently shown that the Orchidaceae is the sister group to all other Asparagales [65-67]. Likewise, Asparagales is the sister group of the Commelinids, a well-supported large clade comprising Arecales (palms), Commelinales (spiderworts), Zingiberales (gingers) and Poales (grasses, sedges and bromeliads). Together, Asparagales and Commelinids occupy a somewhat derived place in the phylogeny of the monocots (Inset, Figure 1). These two groups plus Liliales (lilies), are sister to a clade formed by Pandanales (screwpines) and Dioscoreales (yams). The most basally divergent groups of the monocots are Acorales, Alismatales and Petrosaviaceae, which successively diverged from each other from the group comprised by Asparagales, Commelinids, Liliales, Pandanales and Dioscoreales [65,66].

Here, we present an in-depth phylogenetic analysis of putative class B *DEF*- and *GLO*-like genes from the monocots. In this study, we substantially widen the sample of *DEF- *and *GLO*-like genes, incorporating new sequences from four out of five orchid subfamilies: Vanilloideae, Cypripedioideae, Orchidoideae and Epidendroideae, as well as *Hypoxis*, a member of the Asparagales frequently employed as outgroup in phylogenetic analyses of the Orchidaceae. The molecular phylogenies that we generate are essential for testing hypothesis on the selective regimes that affected class B-like MADS-box genes during the evolution of the orchid perianth. We compare the molecular evolution and expansion of *DEF*- and *GLO*-like genes in the Orchidaceae and order Poales to understand the processes of duplication and natural selection behind the genes associated with the development of the perianths and stamens of the two largest groups of the monocots.

## Results

### Ancient and conserved paralogy in *DEF*-like genes, but not in *GLO*-like genes from orchids

We isolated three to four different cDNA sequences of *DEF*-like genes from each of the orchid species *Vanilla planifolia *(Vanilloideae), *Phragmipedium longifolium *(Cypripedioideae), *Spiranthes odorata *(Orchidoideae) and *Gongora galeata *(Epidendroideae), whereas in *Hypoxis villosa *(Hypoxidaceae), we found only two different sequences. In contrast, in almost all the orchid species analyzed only a single *GLO*-like sequence was identified, with the exception of *Habenaria radiata*, where two *GLO*-like genes have been isolated [61]. In *Hypoxis villosa *two different sequences were found (Table S1 in Additional file 1).

The molecular phylogeny of all monocot *DEF*-like sequences in GenBank (Figure 1) shows strong support for the existence of four orchid-specific clades of orthologous genes that we designate according to the first reported sequence in the literature [57,58]: *PeMADS2*-like genes (clade 1), *OMADS3*-like genes (clade 2), *PeMADS3*-like genes (clade 3) and *PeMADS4*-like genes (clade 4). The phylogenetic reconstruction indicates that clades 1 and 2, as well as clade 3 and 4, are sister clades (Figure 1). The topology of each clade reproduces the phylogeny of the Orchidaceae, indicating that the genes within these clades are orthologues (different genes that originated in speciation events) [42]. The phylogenetic analysis indicates that the sequences in each of the four orchid *DEF*-like clades have features specific to each clade and suggest that, following gene duplication, they might have acquired different functions. In particular, the C-domain of the corresponding proteins is a region possibly involved in protein – protein interactions in multimeric complexes [68]. This region experienced a remarkable level of clade-specific diversification (Additional file 2), whereas in the M-, I- and K-domains most of the substitutions involve amino acids of similar chemical properties. In the positions encoding the MIK-region, nucleotide identity ranges from 68 to 92%, but as expected, it drops to a range between 46 to 90% in the region encoding the C-terminal domain of sequences in clades 1, 3 and 4. This reduced identity is particularly pronounced in clade 2, where it ranges from 28 to 57% as a result of a large number of substitutions (e.g. *PeMADS5 *and *SpodoDEF2*) or early stop codons (e.g. *OMADS3 *and *GogalDEF2*) that eliminated the positions encoding for the otherwise highly conserved C-terminal 'PI-derived' and 'paleoAP3' motifs (Additional file 2). Finally, the monophyletic status of the individual clades of *DEF*-like genes in Orchidaceae (Asparagales) and in the orders Liliales, Poales and Commelinales is supported with posterior probabilities of at least 99% (nodes indicated with arrows, Figure 1). However, the phylogenetic relationships between these clades are undefined by these data.

**Figure 2:**
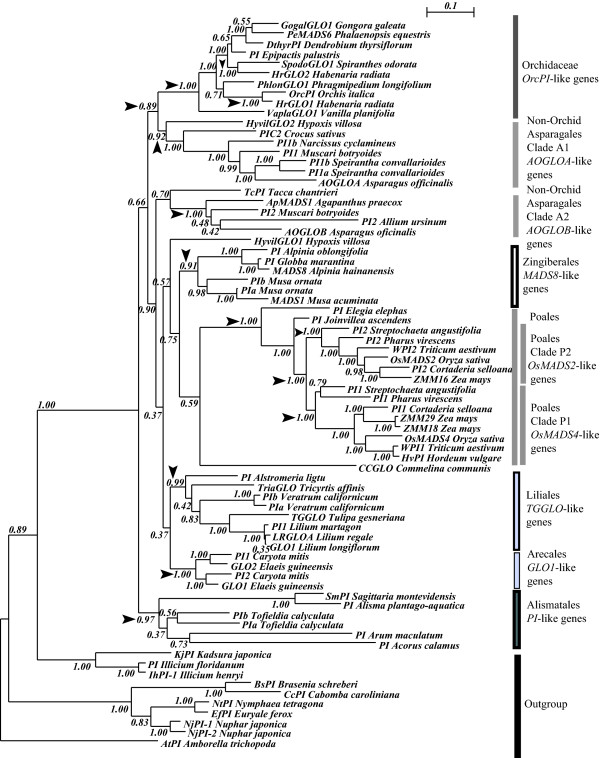
**Phylogeny of monocot *GLO*-like genes based on an alignment of the cDNA encoding all domains**. The figure shows the Bayesian inference phylogeny with all *GLO*-like sequences where the complete coding sequence is available and the new ones reported here. The bars indicate the single clade formed by sequences from the Orchidaceae and of Clades 1 and 2 formed by the rest of the Asparagales, Poales Zingiberales, Liliales, Arecales, Alismatales and the outgroup. The number on every node corresponds to the Bayesian posterior probabilites (PP). Black arrows emphasize nodes that are discussed in the text.

In contrast with the ancient clades of orchid *DEF*-like genes, all *GLO*-like sequences of orchids form a single clade supported with PP = 1.0 (Figure 2). Only *HrGLO1 *together with *OrcPI *and *HrGLO2 *with *SpodoGLO1*, representatives of the subfamily Orchidoideae, form two distinct and equally well-supported subclades (PP = 1.00), one of which appears more closely related to *GLO*-like genes from the subfamily Epidendroideae, whereas the other is closer to the single sequence found in *Phragmipedium longifolium *(Cypripedioideae) (Figure 2). It remains to be seen whether this duplication is specific to the subfamily Orchidoideae or took place after the divergence of the Cypripedioideae and Vanilloideae, as suggested by our analysis. Despite this apparent subfamily-specific duplication and divergence, the sequences of orchid *GLO*-like genes are between 65 to 94% identical at the nucleotide level in the MIK-region and between 75 to 92% in the C-terminal domain, where there are neither large deletions nor early stop codons (Additional file 3). As with, to the clades of orchid *DEF*-like genes, *GLO*-like sequences also reproduce the phylogenetic relationship of the species from which they were isolated.

Each region of the alignments is distinctly variable and thus has different proportions of phylogenetic informative positions. For example, this is reflected in the higher support levels and phylogenetic resolution in the tree obtained with the MIK-region of *DEF*-like genes (Figure 1), compared with the one estimated only with the C-terminal domain or with both regions together (Additional files 4 and 5). This indicates that different domains of these transcription factors are subject to different selective regimes. In contrast, the high degree of conservation in all regions of the *GLO*-like genes is reflected on the relatively similar phylogenetic reconstruction obtained with complete sequences (Figure 2), or individual regions (Additional files 6 and 7).

The apparent lack of family-wide duplications in the *GLO*-like genes within Orchidaceae contrasts with the two well-supported (PP = 0.92 and 1.0, respectively) clades formed by the other Asparagales sequences (Figure 2). According to their species composition, we estimate that these two clades A1 and A2 at least go back to the origin of the Hypoxidaceae and are present in the relatively advanced family Asparagaceae (Figure 2). Most importantly, clade A1 is clearly associated with the corresponding clade of the family Orchidaceae (PP = 0.89).

The grasses belong to an order (Poales) that also shows a well-supported and group-specific internal duplication of their corresponding *GLO*-like genes [63,69]. Furthermore, the clades corresponding to the Liliales, Zingiberales, Arecales and Alismatales are also monophyletic (supported with PPs ≥ 0.91) and contain species-specific gene duplications (Figure 2).

### *DEF*-like genes in clades 1 and 2 of Orchidaceae evolve under different rates of substitution

For every pair of sequences tested, the Maximum Likelihood-based (Relative Rate Test) RRT in HyPhy employs a (Likelihood Ratio Test) LRT to evaluate whether the data fit the null hypothesis of a molecular clock where the branch lengths of a phylogeny are equal or an alternative hypothesis where branch lengths are different.

After adjusting all the results of the RRTs for multiple comparisons, we found that 128 of 231 pairs of orchid *DEF*-like genes rejected the null hypothesis of a constant rate of substitution (P ≤ 0.027). In order to compare the rate differences between the pairs of *DEF*-like sequences that rejected the null hypothesis, we grouped them according to the clade where they belong (Figure 1) and estimated the median of the relative rates of synonymous and nonsynonymous substitution of these groups (Additional file 8). In the case of nonsynonymous substitutions, the most significant comparisons involve species from orchid Clades 1 and 2 with rate between 0.10 and 0.30 substitutions/nucleotide (Additional file 8A). This means that the genes from clade 2 have a rate of *nonsynonymous *substitutions about three times higher than those of sequences in clades 3 and 4 (Additional file 8A). On the other hand, the relative rate of *synonymous *substitution fluctuates independently of the clades compared (Additional file 8B).

In contrast, all 45 pairwise comparisons of orchid *GLO*-like genes failed to reject the null hypothesis (P ≥ 0.05) and have a median rate of nonsynonymous and synonymous substitution of 0.023 and 0.534 substitutions per nucleotide, respectively. Similarly, the RRT with *DEF*- and *GLO*-like genes from Poales did not reject the null hypothesis of constant rates of substitution.

### Purifying selection affected orchid *DEF*- and *GLO*-like genes differently

The analysis of variation of ω along codon sites showed that both *DEF*- and *GLO*-like genes fit a scenario where positive selection is absent (M7, where ω < 1) (Table S2 in Additional file 1). This result is supported by the estimation of ω in *DEF*-like genes with the FEL method from HyPhy. In this analysis, 156 of 249 codons (63%) in the alignment of *DEF*-like genes are under purifying selection with a P ≥ 0.95, whereas the rest of the codons are under neutral evolution (data not shown). However, the tests we applied are conservative and cannot detect positive selection when it affected only a small proportion of sites in a few branches of the phylogeny. For this reason, we implemented a series of tests that focus on specific branches of the phylogenies of *DEF*- and *GLO*-like genes (See "Methods" and Table 1).

**Table 1:** Models of codon substitution employed on the present analysis.

**Level^a^**	**Model**	**Description**	**Site classes**	**Np**	**Free parameters**			**Datasets analyzed^e^**
Sites	M7	Distribution of ω along sites follows a β distribution, no selection	7	2	*p, q*			Alignments of *DEF*- and *GLO*-like genes without basal angiosperm outgroup.
		
	M8	β distribution where ω > 1. Implements BEB^b^.	8	4	*p*_0_, *p, q*, ω_s_, where s = number of site categories			

Branches	M0	one ω value for all branches	0	1	ω			Alignments of *DEF*- and *GLO*-like genes without basal angiosperm outgroup.
		
	M2	n different ω values on n specified branches	0	User-specified	Different ω values corresponding to each of the user-specified groups of branches			

Clades and sites	M1a	nearly neutral evolution	1	2	Clades 1 and 2: 0 < ω_0 _< 1, ω_1 _= 1			Alignment of orchid *DEF*-like genes from clades 1 and 2.
					*p*_0_			
		
	MC	In site classes 2 and 3 selective pressure varies in different parts of the phylogeny. Implements BEB^b^.	2	5	Clade 1: 0 < ω_0 _< 1, ω_1 _= 1, ω_2_			Alignment of orchid *DEF*-like genes from clades 3 and 4.
					Clade 2: 0 < ω_0 _< 1, ω_1 _= 1, ω_3_			Alignment of Poales *GLO*-like genes from clades 1 and 2.
					Proportions: *p*_0_, *p*_1 _^c^			
	
	M3	Assumes several site classes with independently estimated ω.	3	5	ω_0_, ω_1_, ω_2_			Same datasets analyzed with M1a vs. MC.
					Proportions: *p*_0_, *p*_1_,			
		
	MD	In site class 2, selective pressure is different on each clade.	3	6	Clade 1: ω_0_, ω_1_, ω_2 Clade 1_			
					Clade 2: ω_0_, ω_1_, ω_2 Clade 2_			
					Proportions: *p*_0_, *p*_1 _^d^			

Branches and sites	MA1	Neutral or purifying selection on individual codons along specific clades.	4	3	Site class Background Foreground			Alignments of *DEF*- and *GLO*-like genes without basal angiosperm outgroup. Specific branches of the phylogenies were tested in separate analyses.
					0	0 < ω_0 _< 1	0 < ω_0 _< 1	
					1	ω_1 _= 1	ω_1 _= 1	
					2a	0 < ω_0 _< 1	ω_2 _= 1	
					2b	ω_1 _= 1	ω_2 _= 1	
					Proportions: p_0_, p_1 _^d^			
		
	MA	Tests for positive selection on individual codons along specific clades. Implements BEB^b^. Only foreground clades experience positive selection.	4	4	Site class Background Foreground			
					0	0 < ω_0 _< 1	0 < ω_0 _< 1	
					1	ω_1 _= 1	ω_1 _= 1	
					2a	0 < ω_0 _< 1	ω_2_>1	
					2b	ω_1 _= 1	ω_2_>1	
					Proportions: p_0_, p_1 _^d^			

a. Refers to the kind of data where the process of codon substitution is analyzed

b. Bayes Empirical Bayes calculation indicates the posterior probability a site belongs to a given class. This information in valuable to identify sites under positive selection.

c. The values of *p*_0 _and *p*_1 _are also used to calculate *p*_2 _proportions of sites with ω_2 _and ω_3_

d. The values of *p*_0 _and *p*_1 _are used to calculate the proportions of site classes 2a and 2b

e. As previously explained, each dataset includes an un-rooted phylogeny reconstructed with Mr. Bayes using the most appropriate model identified by Modeltest.

**Figure 3:**
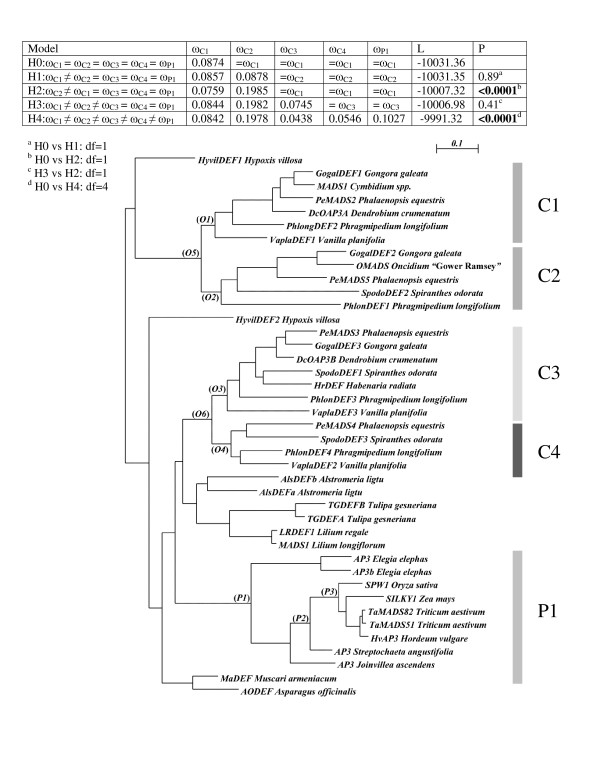
**Test of hypotheses about the variation of ω along the phylogeny of *DEF*-like genes from the Orchidaceae**. The table summarizes the hypotheses tested and their maximum likelihood estimates of ω, which depending on the hypothesis, are free to vary in specific clades (indicated on the phylogeny), while being uniform in the rest of the phylogeny. Column "L" indicates the likelihood value that corresponds to each hypothesis, and P the probability of each model rejecting its corresponding null hypothesis in the series of LRTs indicated at the bottom of the table. The phylogeny employed in these tests is based on the alignment of the complete coding sequences of *DEF*-like genes. However, to facilitate the inference of the parameters for each model, the outgroup and sequences from clades not directly relevant to the analyses were removed from the alignment and the phylogeny was re-estimated with an *ad hoc *model as described in *Methods*. The branches labeled with italics were tested for positive selection with branch-site models A1 and A.

To determine whether the ω ratio varies substantially in clades of *DEF- *and *GLO*-like genes from the order Poales and the family Orchidaceae we compared the null hypothesis (H0) of a "single ω ratio for all branches" (M0) with several nested alternative hypotheses (H#) represented by M2, where one or more clades of interest are free to adopt different values estimated from the data (Figure 3).

In the case of *DEF*-like genes, we tested five hypotheses where H0 assumes that ω adopted the same value along all branches of the phylogeny. Using LRT we compared this scenario with four relevant alternative hypothesis: H1, where a specific ω is estimated for the branches of clade 1 (C1); H2, where clade 2 (C2) has a different ω to the rest of the branches; H3, where orchid clades C1, C2 and C3 have estimated ω values different to the rest of the branches and H4, where the branches of each of the four clades of the orchid *DEF*-like genes and the single clade of Poales *DEF*-like genes (P1) have ω estimates significantly different from the rest of the branches (Figure 3).

Hypotheses H0 and H3, which assume no significant differences between all or some of the groups of branches tested, are significantly (≤ 0.0001) rejected in favor of H4, where specific ω values were estimated for the different groups of *DEF*-like genes from Orchidaceae and Poales (Figure 3). Most importantly, in hypothesis H4 where ω is estimated for each group of branches, these values converge and indicate that although all clades tested are under strong purifying selection, the group of branches in orchid clade C2 has an ω value of 0.1978, which is more than twice those estimated for other groups of branches (Figure 3).

**Figure 4:**
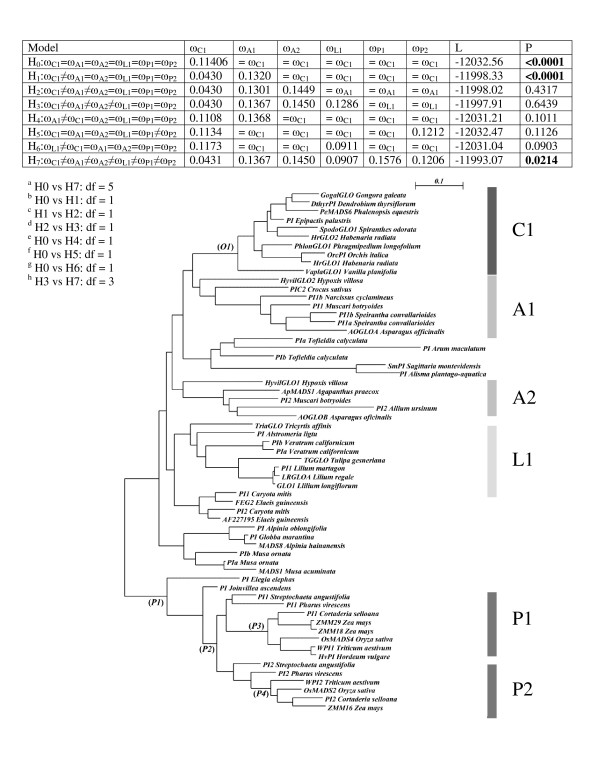
**Test of hypotheses about the variation of ω along the phylogeny of *GLO*-like genes from the Orchidaceae**. This figure is organized like Figure 3, but the phylogeny employed in these tests is based on the alignment of the complete coding sequences of *GLO*-like genes. However, to facilitate the inference of the parameters for each model, the outgroup and sequences from clades not directly relevant to the analyses were removed from the alignment and the phylogeny was re-estimated with an *ad hoc *model as described in *Methods*. The branches labeled with italics were tested for positive selection with branch-site models A1 and A.

Similarly, in the corresponding analysis of monocot *GLO*-like genes we tested a null hypothesis H0 where all clades have the same estimated ω value and six alternative hypotheses where this rate is estimated for one or more relevant groups of branches. To determine whether gene duplication is associated with a distinct pattern of evolution, we estimated ω on both the single clades of *GLO*-like genes from orchids (C1) and Liliales (L1) as well as the paralogous clades from Poales (P1, P2) and the remainder of Asparagales (A1, A2) (Figure 4).

Comparisons of H0 vs H1 and H7 rejected the scenario where ω is the same in all branches, thus favoring the alternative hypotheses where the orchid clade has ω = 0.043 (*P *≤ 0.0001) (Figure 4). Like the *DEF*-like sequences, all clades of *GLO*-like genes are under strong purifying selection, but this is especially pronounced in the case of the single orchid clade (Figure 4).

We complemented the previous branch-based analysis with two tests for detecting rate shifts *in *paralogous clades [70]. Specifically, we tested paralogous clades of *DEF*-like genes 1 and 2 as well as 3 and 4 and the *GLO*-like clades P1 and P2 (Tables 2, 3 and 4). The tests showed that in clades 1 and 2 there have been significant (P ≤ 0.0005) shifts in the value of ω following gene duplication (Table 2). Both models MC and MD estimated that approximately 42% of the sites of both clade 1 and 2 have divergent ω values, (ω_2 C1 _= 0.21 and ω_2 C2 _= 0.50, respectively), and that these site classes are on a background of stronger purifying selection (ω_1 _= 0.033 in 55% of the sites) and neutral evolution (ω_0 _= 1.28 or ω_1 _= 1.0 in approximately 2% of the sites) (Table 2). These results are consistent with those estimated by the previous analysis with M2, where clade 1 is under more stringent purifying selection than clade 2 (Figure 3). Also in agreement with the previous analysis of M0 vs. M2, the analysis of *DEF*-like genes in clades 3 and 4 showed that 75% of their sites are under stronger purifying selection (ω_2 C4 _= 0.025 and ω_2 C3 _= 0.017) than genes in clades 1 and 2 (Table 3).

**Table 2:** Parameter estimates and LRT of M3 vs. MD and M1a vs. MC in *DEF*-like genes from Orchidaceae-specific clades 1 and 2^1^.

**Model**	**Estimate of parameters**	**L**
M3 discrete k = 2	ω_0 _= 0.04544, f_0 _= 0.63768 ω_1 _= 0.42599, f_1 _= 0.36232	-3516.356560

M3 discrete k = 3	ω_0 _= 0.04544, f_0 _= 0.63768 ω_1 _= 0.42599, f_1 _= 0.36232 ω_2 _= 38.73814, f_2 _= 0.00000	-3516.356560

Model D k = 2	ω_0 _= 0.04102, f_0 _= 0.60628 ω_1C1 _= 0.26333, ω_1C2 _= 0.59513, f_1_= 0.39372	-3508.313165

**Model D** **k = 3**	**ω_0 _= 1.28841, f_0 _= 0.02038** **ω_1 _= 0.03309, f_1 _= 0.55669** **ω_2C1 _= 0.21766, ω_2C2 _= 0.50952, f_2 _= 0.42294**	**-3506.393271**

Model 1a	ω_0_= 0.09066, f_0 _= 0.75319 ω_1 _= 1.00000, f_1 _= 0.24681	-3540.435701

**Model C**	**ω_0 _= 0.03251, f_0 _= 0.55437** **ω_1 _= 1.00000, f_1 _= 0.02680** **ω_2C1 _= 0.21104, ω_2C2 _= 0.50104, f_2 _= 0.41883**	**-3506.594944**

LRTs of variable ω's among sites

M3(k = 2) vs M3 (k = 3) 2δ = 3.617532 df = 2 P = 0.1639

LRTs of variable ω's among sites and branches

M3 (k = 2) vs MD (k = 2) 2δ = 16.08679 df = 2 **P = 0.0003**

M3 (k = 3) vs MD (k = 3) 2δ = 16.309046 df = 2 **P = 0.0003**

M3 (k = 2) vs MD (k = 3) 2δ = 19.926578 df = 4 **P = 0.0005**

M1a vs MC 2δ = 67.681514 df = 2 **P < 0.0001**

1. Bonferroni correction for the 5 tests applied to each pair of clades: 0.008333

**Table 3:** Parameter estimates and LRT of M3 vs MD and M1a vs MC in *DEF*-like genes from Orchidaceae-specific clades 3 and 4^1^.

**Model**	**Estimate of parameters**	**L**
M3 discrete k = 2	ω_0 _= 0.02182, f_0 _= 0.76519 ω_1 _= 0.23442, f_1 _= 0.23481	-3729.728309

M3 discrete k = 3	ω_0 _= 0.02182, f_0 _= 0.09116 ω_1 _= 0.02182, f_1_= 0.67403 ω_2 _= 0.23442, f_2 _= 0.23481	-3729.728309

Model D k = 2	ω_0 _= 0.02175, f_0_= 0.76410 ω_1C4 _= 0.24611, ω_1C3 _= 0.22536, f_1_= 0.23590	-3729.649245

Model D k = 3	ω_0 _= 0.23556, f_0 _= 0.23301 ω_1 _= 0.01603, f_1 _= 0.00000 ω_2C4 _= 0.02665, ω_2C3 _= 0.01863, f_2 _= 0.76699	-3729.138799

Model 1a	ω_0 _= 0.05538, f_0 _= 0.95421 ω_1 _= 1.00000, f_1 _= 0.04579	-3764.994659

**Model C**	**ω_0 _= 0.21797, f_0 _= 0.23733** **ω_1 _= 1.00000, f_1 _= 0.00687** **ω_2C4 _= 0.02599, ω_2C3 _= 0.01796, f_2 _= 0.75580**	**-3728.737317**

LRTs of variable ω's among sites

M3(k = 2) vs M3 (k = 3) 2δ = 0 df = 2 P = 1

LRTs of variable ω's among sites and branches

M3 (k = 2) vs MD (k = 2) 2δ = 0.158128 df = 2 P = 0.9240

M3 (k = 3) vs MD (k = 3) 2δ = 1.17902 df = 2 P = 0.5546

M3 (k = 2) vs MD (k = 3) 2δ = 1.17902 df = 4 P = 0.8815

M1a vs MC 2δ = 72.514684 df = 2 P < 0.0001

1. Bonferroni correction for the 6 tests applied to each pair of clades: 0.008333

**Table 4:** Parameter estimates and LRT of M3 vs. MD and M1a vs. MC in *GLO*-like genes from Poales-specific clades 1 and 2^1^.

**Model**	**Estimate of parameters**	**L**
M3 discrete k = 2	ω_0 _= 0.01674, f_0 _= 0.58033 ω_1 _= 0.22754, f_1 _= 0.41967	-3440.487607

M3 discrete k = 3	ω_0 _= 0.01674, f_0 _= 0.58033 ω_1 _= 0.22754, f_1 _= 0.41967 ω_2 _= 32.14375, f_2 _= 0.00000	-3440.487645

Model D k = 2	ω_0 _= 0.22754, f_0 _= 0.41968 ω_1P1 _= 0.01605, ω_1P2 _= 0.01734, f_1 _= 0.58032	-3440.475354

Model D k = 3	ω_0 _= 48.10696, f_0 _= 0.00000 ω_1 _= 0.01695, f_1 _= 0.58176 ω_2P1 _= 0.26322, ω_2P2 _= 0.20131, f_2 _= 0.41824	-3439.561525

Model 1a	ω_0 _= 0.08103, f_0 _= 0.96439 ω_1 _= 1.00000, f_1 _= 0.03561	-3484.524831

**Model C**	**ω_0 _= 0.21164, f_0 _= 0.42529** **ω_1 _= 1.00000, f_1 _= 0.01051** **ω_2P1 _= 0.01484, ω_2P2 _= 0.01608, f_2 _= 0.56420**	**-3439.651822**

LRTs of variable ω's among sites

M3(k =) vs M3 (k =) 2δ = -7.6E-05 df = 2 P = 1

LRTs of variable ω's among sites and branches

M3 (k = 2) vs MD (k = 2) 2δ = 0.024506 df = 2 P = 0.9878

M3 (k = 3) vs MD (k = 3) 2δ = 1.85224 df = 2 P = 0.3961

M3 (k = 2) vs MD (k = 3) 2δ = 1.852164 df = 4 P = 0.7629

M1a vs MC 2δ = 89.746018 df = 2 P < 0.0001

1. Bonferroni correction for the 6 tests applied to each pair of clades: 0.008333

A similar set of branch-site tests used to study the divergence of the two clades of Poales *GLO*-like genes (Table 4) showed that MC, the model where shifts in ω take place after gene duplication, fits the data significantly better than other hypotheses (*P *< 0.0001). This result confirms that although P1 and P2 are under purifying selection (ω_0_= 0.211) as previously shown with M0 vs M2, ω is especially stringent when considering the clades individually (ω_2P1 _= 0.0148 and ω_2P2 _= 0.01608, respectively) (Table 4).

### Positive selection preceded diversification of *DEF*-like genes in Poales

We estimated ω in the branches preceding the origin and subsequent duplications of *DEF*- and *GLO*-like genes from Orchidaceae and Poales by employing the branch-site models MA and MA1 (Figure 3, Table 5). As discussed in "Methods", MA and MA1 assume that branches in a phylogeny are divided in foreground and background while their codon sites are sorted in classes 0, 1, 2a and 2b according to their ω value (Table 1). In site classes 0 and 1, both foreground and background branches have 0 < ω_0 _< 1 or ω_1 _= 1, respectively. However, in the foreground branches, MA assumes that site classes 2a and 2b have ω_2 _> 1 whereas in MA1 these site classes have ω_2 _= 1 (Table 1). Therefore, comparing MA with MA1 is a test of positive selection on branches and sites.

**Table 5:** Parameter estimates and LRT of MA1 vs. MA in *DEF*-like genes from Poales.

**Clade**	**Model**	**Estimate of parameters**	**Positive selection**	**L**
Poales (*P1*)	A	ω_0 _= 0.08303, f_0 _= 0.89319 ω_1 _= 1.00000, f_1 _= 0.03486 ω_2a fore =_936.29115, ω_2a back =_0.08303, f_2a _= 0.06925 ω_2b fore =_936.29115, ω_2b back =_1.00000, f_2b _= 0.00270	45 L 0.612 50 S 0.703 54 L 0.531 55 S 0.996** 77 I 0.556 82 A 0.588 86 K 0.583 89 N 0.991** 92 N 0.517 103 K 0.689 116 I 0.573 117 K 0.976* 126 L 0.625 132 L 0.993** 152 V 0.604 159 H 0.535 164 R 0.899← 167 E 0.742 170 N 0.931← 175 E 0.639 177 Y 0.996** 181 L 0.610	-9975.021057
	
	A1	ω_0 _= 0.08196, f_0 _= 0.78512 ω_1 _= 1.00000, f_1 _= 0.03088 ω_2a fore =_1.00000, ω_2a back =_0.08196, f_2a _= 0.17704 ω_2b fore =_1.00000, ω_2b back =_1.00000, f_2b _= 0.00696		-9979.237754

LRT	2δ = 8.433394 df = 1 P = 0.0037			

Poales (*P2*)	A	ω_0 _= 0.08281, f_0 _= 0.86235 ω_1 _= 1.00000, f_1 _= 0.03283 ω_2a fore =_4.43236, ω_2a back =_0.08281, f_2a _= 0.10097 ω_2b fore =_4.43236, ω_2b back =_1.00000, f_2b _= 0.00384	14 S 0.813 42 E 0.747 44 S 0.846 50 S 0.993** 54 L 0.571 57 F 0.799 64 T 0.787 75 S 0.992** 78 N 0.706 81 S 0.788 103 K 0.893 128 E 0.892 142 N 0.821 154 N 0.736 171 Y 0.801	-9983.412368
	
	A1	ω_0 _= 0.08278, f_0 _= 0.76740 ω_1 _= 1.00000, f_1 _= 0.02922 ω_2a fore =_1.00000, ω_2a back =_0.08278, f_2a _= 0.19592 ω_2b fore =_1.00000, ω_2b back =_1.00000, f_2b _= 0.00746		-9984.514801

LRT	2δ = 2.204866 df = 1 P = 0.1376			

Poales (*P3*)	A	ω_0 _= 0.08393, f_0 _= 0.95687 ω_1 _= 1.00000, f_1 _= 0.03593 ω_2a fore =_8.40943, ω_2a back =_0.08393, f_2a _= 0.00694 ω_2b fore =_8.40943, ω_2b back =_1.00000, f_2b _= 0.00026	33 T 0.517 183 L 0.954*	-9987.927062
	
	A1	ω_0 _= 0.08401, f_0 _= 0.95276 ω_1 _= 1.00000, f_1 _= 0.03571 ω_2a fore =_1.00000, ω_2a back =_0.08401, f_2a _= 0.01111 ω_2b fore =_1.00000, ω_2b back =_1.00000, f_2b _= 0.00042		-9988.396587

LRT	2δ = 0.93905 df = 1 P = 0.3325			

The branches tested are those labeled with cursive fonts in Figure 3.

**Figure 5:**
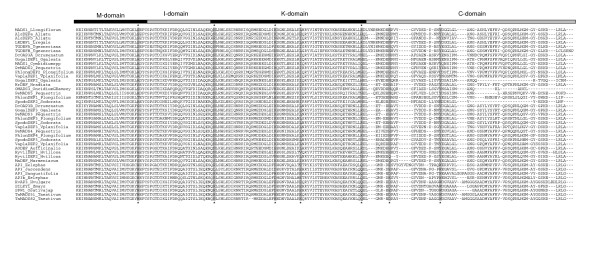
**Sites under positive selection in Poales, mapped on the alignment of monocot *DEF*-like proteins containing only all variable amino-acid positions**. The sites with a posterior probability of ≥ 0.90 listed in Table 5 are marked with an asterisk on the alignment. Although the sequences under positive selection correspond to the Poales, the rest of the sequences in the alignment are included for a better comparison.

The branch-site analysis showed positive selection on the branch preceding the divergence of *DEF*-like genes in the Poales (Table 5). In this branch, the corresponding Bayes Empirical Bayes (BEB) analysis, included in MA, detected seven codon sites under positive selection (PP ≥ 0.90), most of which are in the region encoding the K-domain (Figure 5). The codon substitutions in these sites represent both radical and preferential changes in the chemical properties of the amino acids that they encode (Figure 5). The positions under positive selection that we detected in this lineage are different from those identified by Hernández-Hernández et al. (2007) [22] in other nodes of the phylogeny of class B genes as well as from sites in AP3 from *Arabidopsis thaliana *that are critical for protein – protein interactions [71].

Furthermore, we tracked the occurrence of positive selection along the branches representing the divergence of *DEF*-like genes in Poales following the emergence of *E. elephas *and *S. angusifolia *(Table 5, Figure 3). Although in these analyses the foreground branches have ω > 1 and sites under positive selection were identified, the LRT does not reject the null model MA1.

Similarly, we tested for positive selection in branches that in Orchidaceae precede and follow the duplications of *DEF*-like clades 1, 2, 3 and 4 (Figure 3, Table S3 in Additional file 1). The LRTs of these analyses did not reject the null hypothesis where over 95% of the codon sites are under purifying selection (ω_0 _= 0.084).

Further analyses in different nodes of the evolution of *GLO*-like genes in the Orchidaceae and Poales did not reject the null hypothesis where most of the sites have ω_0 _= 0.112 (Figure 4 and Table S4 in Additional file 1).

## Discussion

The phylogeny of MADS-box genes indicates that functional classes A, B, C+D and E of floral homeotic genes are grouped in distinct clades [23-25], suggesting that duplication and functional diversification of MADS-box genes contributed significantly to the evolution of the flower structure [23,33,72,73]. Thus, studying the phylogeny of MADS-box genes is a powerful tool to better understand the evolution of plant morphology [23,33,73-75]. Here we investigate the molecular evolution of putative class B genes from monocots, with special emphasis on *DEF- *and *GLO*-like genes from orchids and Poales. We have chosen these genes because they most likely specify the identity of both perianth organs and stamens throughout the angiosperms, and they are thus essential for understanding important aspects of the morphological divergence of the orchids and grasses from the rest of the monocots [41,42,63,76,77]. Specifically, we determined whether shifts in the rates and patterns of nucleotide substitution of duplicated class B genes are correlated with their subsequent functional divergence and thus with the evolution of novel perianth structures in orchids and grasses.

### Ancient duplication of *DEF*-like genes possibly facilitated the diversification of the orchid flower morphology

The phylogenetic analyses presented here strongly support the existence of four ancient, orchid-specific clades of *DEF*-like genes. These four clades and their internal branches are generally supported with PP values > 0.99. Our data strongly suggest that the sequences within each clade are orthologues, because they reproduce the systematic relationships reported for the four most derived subfamilies of the Orchidaceae: (Vanilloideae(Cypripedioideae(Orchidoideae, Epidendroideae))) [44,78]. This indicates that the clades are the result of ancient gene duplication events that at least precede the origin of the subfamily Vanilloideae, which according to recent estimates, emerged between 71 to 62 MYA [79]. The ancient origin, conservation and unique expression (see below) of these distinct groups of orchid-specific genes suggest that they have key roles in determining the organ identities behind the characteristic orchid floral morphology [42,62]. Our data show that clades 1 and 2, as well as clades 3 and 4, are sister to each other. Although clades 1 and 2 seem most closely related to some other *DEF*-like genes in the Asparagales (Figure 1), the data available do not allow unequivocal reconstruction of the deeper phylogenetic relationships between orchid paralogous groups and the rest of the monocot sequences.

Exceptionally, we found that members of subfamily Orchidaceae have two lineages of *GLO*-like genes (Figure 2). We argue that these two lineages may be the result of a subfamily-specific duplication because exhaustive RACE on several cDNA pools from species in subfamilies Vanilloideae (*Vanilla planifolia*), Cypripedioideae (*Phragmipedium longifolium*) and Epidendroideae (*Gongora galeata*) yielded only a single *GLO*-like gene. The fact that *GLO*-like genes from Cypripedioideae and Epidendroideae seem to associate with different lineages of Orchidoideae (Figure 2), may be the result of differential gain and loss of duplicate genes during the evolution of these subfamilies.

The phylogeny of *DEF*-like genes provides the evolutionary context needed to interpret functional information on these sequences and suggests a particular model of perianth organ determination and evolution [42,62]. Mapping the known expression patterns onto the phylogenies of orchid *DEF*-like sequences shows that genes belonging to the same clade have the same or very similar expression domains [42]. These clade-specific expression patterns suggest that the duplication events that gave rise to the ancestors of these clades were followed by transcriptional and functional differentiation of each paralogue.

Specifically, in addition to expression in the gynostemium, genes from both clades 1 and 2 are expressed in the outer and inner tepals, whereas the expression of genes in clades 3 and 4 is limited to the inner tepals or to the lip, respectively [42]. Considering both the expression patterns of each clade and the events of gene duplication that generated them, it seems likely that after the first gene duplication, the ancestor of genes in clades 3 and 4 produced the differences between inner (expression "on") and outer tepals (expression "off"). The resulting morphology might still exist in the basal genus *Apostasia *(subfamily Apostasioideae) whose flowers do not yet possess elaborate lips [45]. Similarly, the distinction between lateral inner tepals and the lip emerged after a second gene duplication affecting the ancestor of clades 3 and 4, followed by changes in the *cis*-regulatory regions of the duplicated genes that resulted in differential expression and determination of lip identity by clade 4 genes [42]. Validating this scenario involves further characterization of the patterns of expression of *DEF*- and *GLO*-like genes in the basal and relatively species-poor families Apostasioideae, Vanilloideae and Cypripedioideae.

Although genes in clade 1 and 2 are expressed in all the perianth organs, it is unlikely that they have a completely redundant role in the determination of perianth organ identity. According to our phylogenetic reconstruction, these clades were already present in the Vanilloideae, an orchid subfamily that emerged at least 62 million years ago [79]. Retention of duplicate genes for such a long time is alone a strong argument for functional diversification. Furthermore, our analyses show that the members in these clades have substantial differences in their C-terminal domains, distinct rates of nonsynonymous substitution and significant differences in their respective patterns of purifying selection (Figures 1, 3, Tables 2, 3, Additional file 2). Specifically, in the sequences of clade 2 *DEF*-like genes, a relatively high proportion of non-synonymous substitutions followed the duplication that generated this clade and eventually caused the truncations in the open reading frames that characterize the sequences analyzed here. Despite the high level of divergence of clade 2 orchid *DEF*-like genes, our detailed characterization of ω along the phylogeny of all orchid class B genes did not indicate specific branches or sites where positive selection took place. Our estimation of ω along sites, branches and specific clades documents a scenario of prevalent purifying selection (Figures 3, 4, Tables 2 to 4) that agrees with previous analyses of class B genes and other floral organ identity genes [13,14,22,80]. However, we cannot completely rule out the occurrence of positive selection, since its effects on few sites during a brief evolutionary period can be masked by ensuing and continuous purifying selection [81,82].

The uniform expression of *GLO*-like genes in the perianth organs of orchids [59-61], indicates that although these genes are probably essential for proper flower development, they may not play a role in determining the different organ identities in the orchid perianth [42]. However, further work is needed to determine whether the subfamily Orchidoideae-specific gene duplication (Figure 2) is associated with differential patterns of expression and function and thus with the specific morphology of this subfamily.

Similarly to *DEF*-like sequences, all clades of *GLO*-like genes were under strong purifying selection, but this is especially pronounced in the case of the single clade of genes from orchids (Figure 4). We think the differences in the ω ratio reflect the distinct selective constraints affecting duplicated class B genes. Specifically, the ratios corresponding to the generally monogenic clades of the Orchidaceae (ω = 0.043) and Liliales (ω = 0.0907) are lower than those of the rest of the Asparagales and the Poales, where there are two *GLO*-like loci (ω = 0.1206 and ω = 0.157, respectively). We reasoned that in orchids, and to a lesser extent in *GLO*-like sequences from Liliales, the lower rate of substitution reflects the stable co-evolutionary interaction between the product of a single *GLO*-like gene with several DEF-like interaction partners. Also, duplicated loci in clades A1 and A2 from the rest of the Asparagales, as well as P1 and P2 from the Poales, may be completely or partially redundant and thus under less stringent purifying selection than their single-copy homologues in other species (Figure 4).

The fact that each of the clades of *DEF- *and *GLO*-like sequences replicates the systematic relationships of the four most advanced subfamilies of the Orchidaceae [44,78] suggests that these sequences may be useful for reconstructing the phylogeny of the Orchidaceae, provided the analysis exclusively involves orthologous sequences.

### Positive selection in *DEF*-like genes from Poales preceded the evolution of the grass floret

The order Poales contains 18 families, of which three are represented in our analyses of *DEF*-like genes: Restionaceae (*Elegia elephas*), Joinvilleaceae (*Joinvillea ascendens*) and Poaceae with the rest of the species (*Streptochaeta angustifolia*, *Oryza sativa, Triticum aestivum, Hordeum vulgare, Zea mays*). Recent phylogenetic analyses showed that the early-diverging lineage of the Restionaceae is the sister group of a clade containing Joinvilleaceae and the more derived Poaceae; the latter comprising most of the species of Poales [47,65]. Previous studies [63] sampled species representing the morphological transition from the typical monocot flower to the grass floret. Specifically, the flowers of the basal *Elegia elephas *and *Joinvillea ascendens *are actinomorphic, possessing two trimerous whorls of tepals and three or six stamens, respectively [63]. In contrast, the floret of the early-diverging grass *Streptochaeta angustifolia *has 12 bracts. The trimerous arrangement of the six bracts VII to XII have been interpreted as the first and second perianth whorls [63]. Most importantly, the expression of class B gene *SaAP3 *in the last three bracts and in the six stamens suggests that these bracts are second whorl organs, possibly a transitional form preceding the evolution of actual lodicules [63,83]. Moreover, the study of Whipple et al (2007) suggests that class B genes control the identity of second whorl organs in a broader sense than only petal identity. The morphologies outlined above contrast with the grass floret found in the rest of the Poaceae species analyzed here. Specifically, the floret is formed by one lemma and one palea subtending a flower formed by two or three lodicules, and the male and female reproductive organs. The grass floret is also a zygomorphic structure like the orchid flower, mostly due to frequent differential suppression of stamens from different whorls [84,85] and suppression of the adaxial lodicule in most derived grasses (e.g. *Hordeum, Oryza*) [48,84].

In this context, the evidence for positive selection in the branch that represents the divergence of Poales *DEF*-like genes from the rest of the monocot genes (Figure 3, Table 5) suggests that during the morphological divergence of grasses a series of nonsynonymous substitutions took place before the emergence of the characteristic grass floret. Positive selection probably continued along the branches following the divergence from *Elegia elephas *and *Streptochaeta angustifolia *(Table 5), but the corresponding signal eventually became masked by positions under purifying selection that probably encode amino acids essential for a grass-specific network of class B gene targets. The facts that class B transcription factors SILKY1 (DEF-like) and ZMM16 (GLO-like) from maize share conserved heterodimerization specificity with *Arabidopsis thaliana *class B proteins APETALA3 and PISTILLATA *in vitro *and rescue the corresponding null mutants makes it conceivable that the residues mediating the interaction between these two sets of class B proteins already existed in the most recent common ancestor of monocots and eudicots [76]. This not only suggests that the mechanisms of organ identity determination for second whorl organs were established early during the evolution of the angiosperms, but also that the subsequent morphological divergence in the grasses is probably associated with the lineage-specific substitutions in the K-domain that we detected. Substitutions in this domain may have the potential for changing higher order complex formation of class B proteins and thus their binding specificity to downstream target genes, eventually enabling them to coordinate the development of novel perianth structures.

Methods for detecting positive natural selection, like the ones we employed here, are powerful tools to generate and experimentally verify interesting hypothesis on the evolution of new gene functions and phenotypes. Recently, molecular adaptation of polygalacturonase inhibitor protein, TRIM5α, feruloyl esterase A and salicylic acid methyltransferase has been tested on proteins encoded by genes where nucleotides under positive selection were modified via site-directed mutagenesis or by domain-swapping experiments generating genes encoding chimeric proteins [86-89]. In the future, similar approaches could be employed to assess the functional consequences of positive selection in DEF-like transcription factors from Poales. For example, hypothesis on the transcriptional activity of these proteins could be evaluated by substituting sites under positive selection with the ones found in present-day orchids or the ones inferred to have existed in their common ancestor. The interactions of chimeric or mutagenized DEF-like proteins from Poales with other MADS-domain transcription factors and DNA could be assayed *in vitro *via yeast-two-hybrid and electrophoretic mobility shift assays [90], respectively, or *in planta *employing Fluorescence Resonance Energy Transfer (FRET) [91].

### Functional consequences of C-terminal deletions in clade 2 DEF-like proteins of orchids

Previous phylogenetic analyses of the DEF- and GLO-like proteins identified specific motifs characteristic for certain plant lineages [33,35]. Similarly to what we observed in the different lineages of orchid *DEF*-like genes, most of these characteristic motifs evolved in the C-terminal domain of the encoded proteins. This domain is highly variable and in class B proteins it might be involved in the formation of multimeric transcription factor complexes [39,92]. The evolutionary importance of these differences in the C-terminal domain is supported by their long evolutionary conservation (Additional file 2). In particular, the *family specific *C-terminal deletions observed in orchid DEF-like proteins from clade 2 are exceptional if one considers that in all published DEF-like proteins, C-terminal deletions are only *species specific *(M. Mondragón-Palomino, unpublished results). The importance of these orchid-specific deletions is highlighted by recent findings on the occurrence of frameshift mutations in the regions encoding C-terminal motifs of the members of the clades of *DEF- *and *GLO-*like genes [93,94]. For example, the study by Kramer et al[94] indicates that the C-terminal motif 'euAP3' resulted from a translational frameshift caused by a single nucleotide deletion in the ancestral motif 'paleoAP3'. Because the paleoAP3 motif is found in DEF-like proteins throughout the angiosperms (e.g. in TM6-like proteins of eudicots), whereas the eu*AP3 *motif is found only in DEF-like proteins of higher eudicots, Lamb and Irish [95], suggested that there is a causal relationship between duplication of *DEF*-like genes, mutations in the C-domain, functional differentiation of *DEF*-like genes and the emergence of specific floral morphologies. Although the previous evidence argues for the functional importance of the C-terminal deletions in the orchid clade 2 proteins, there is surprisingly little experimental evidence for a function of the C-terminal domain, not only in orchids but also in other species. Specifically, recent independent studies with truncated DEF-like proteins from *Arabidopsis thaliana *and *Chloranthus spicatus *suggest that the C-terminal domain is not essential for floral identity [96,97]. Interestingly, yeast-two hybrid experiments involving OMADS3 from orchid *Oncidium *"Gower Ramsey", a clade 2 protein lacking the conserved C-terminal motifs PI-derived and paleoAP3 (Additional file 2), showed that this protein forms homodimers [57], opening up the possibility of novel regulatory functions for the proteins of clade 2. However, results derived from experiments using DEF-like proteins with truncated C-terminal domains of other monocots do not lend support to a particular role of this domain in dimerization [68,90].

## Conclusion

**Figure 6:**
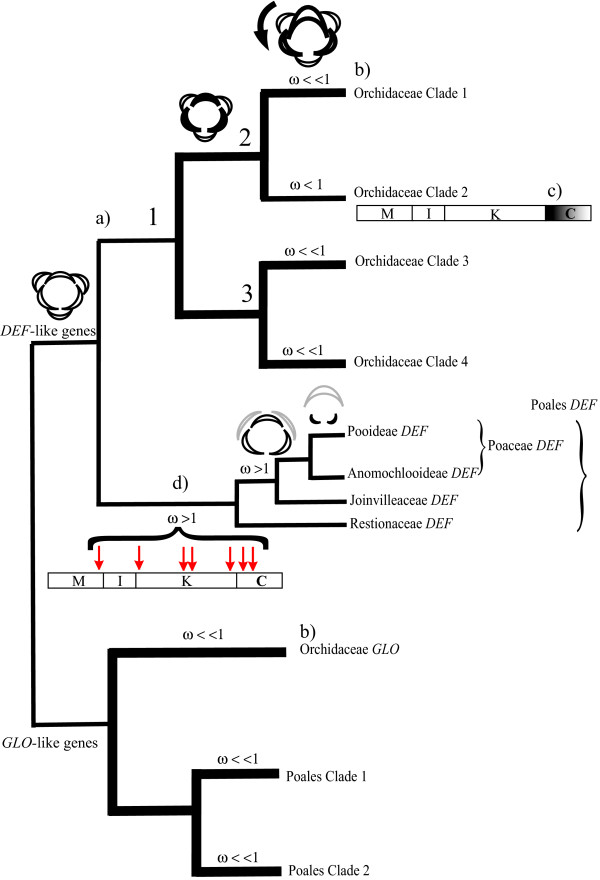
**Trends in the molecular evolution of *DEF*- and *GLO*-like genes from Orchidaceae and Poales**. In this schematic phylogeny the major morphological and evolutionary features of the two lineages of class B genes from Orchidaceae and Poales are summarized: (a) Three gene duplications resulted in four *DEF*-like genes in the Orchidaceae. The points where these events happened are labeled 1 to 3 without assuming any specific order for duplication events 2 and 3. This includes the reasonable possibility that duplications 2 and 3 occurred simultaneously (e.g. by partial or complete genome duplication). We propose that duplication of *DEF*-like genes is associated to the emergence of new perianth organ identities. Specifically, from an ancestor with an actinomorphic perianth evolved after the first round of *DEF*-like gene duplication an intermediate morphology where inner and outer tepals were different. Following a final round of duplication and functional divergence of clade 4 genes the lip or labellum organ identity emerged and made the perianth zygomorphic. The arrow indicates a 180° rotation of the pedicel and or ovary (resupination) that in most orchids sets the lip, the developmentally uppermost tepal, in a lowermost portion of the perianth. (b) Purifying selection prevails in *DEF*- and *GLO*-like genes (ω << 1), but is less stringent in Orchidaceae *DEF*-like genes from clade 2 (ω < 1); (c) Some proteins encoded by *DEF*-like genes from clade 2 miss part of the C-terminal domain; (d) Positive selection (ω>1) took place at the base of the Poales *DEF*-like genes. Most of the positions involved are in the K-domain. Positive selection was also detected, although not statistically significant, on the lineage linking *DEF*-like genes from Restionaceae with the rest of the Poales. The highly reduced and zygomorphic perianth or grasses evolved from a common ancestor with the "typical" monocot perianth morphology. Here represented by black half-moons are the inner perianth organs of *Streptochaeta angustifolia *(Anomochlooideae) and *Oryza sativa *(Pooideae) to illustrate the morphological transition that was preceded by the process of positive selection here documented. In this diagram the palea is in gray and the outer perianth whorl is absent.

Our results strongly support the existence of four distinct paralogous clades of orchid *DEF*-like genes that originated at least 62 MYA via three gene duplications. It appears likely that the gene duplication that gave rise to the ancestors of clades 1 and 2 on the one hand and clades 3 and 4 on the other hand occurred simultaneously, due to a whole genome duplication (WGD) (Figure 6). In contrast, all orchid *GLO*-like genes form a single, highly conserved clade where subfamily-specific gene duplicates may only have been retained in Orchidoideae. Our analyses show that the four clades of orchid *DEF*-like genes are significantly distinct in their level of sequence divergence, strength of purifying selection and clade-specific sequence motifs. These differences, together with the clade-specific patterns of gene expression [42,57,58,60-62], suggest that after duplication, changes in the promoter and coding region resulted in sub- and neofunctionalization of the paralogous *DEF*-like genes. We argue that the orchid *DEF*-like genes acquired new functions in the specification of at least three different orchid-specific perianth organ identities by changes in expression patterns and target gene recognition. According to the 'orchid code' [42,62], in addition to the expression of a *GLO*-like gene and possibly also *AP1*- and/or *SEP*-like genes, the combined expression of clade 1 and clade 2 genes in the first floral whorl determines the formation of outer tepals. In the second whorl, the identity of lateral inner tepals is determined by the combined action of clade 1, clade 2 and clade 3 genes. The identity of the lip is determined by the organ-specific expression of a clade 4 gene in addition to the expression of all the other *DEF*-like genes [42]. The neofunctionalization of the four orchid *DEF*-like genes, implicit in the 'orchid code' may have been a crucial event in the morphological diversification of the orchid flower (Figure 6). It could have 'modularized' the orchid perianth, by enabling an individual response of every type of organ to natural selection and thus independent evolution [62]. Contrary to the case in lilies and tulips, where perianth development is very likely under control of one set of floral homeotic genes, including *DEF*-like and *GLO*-like genes [54], the different perianth organs of the orchids are controlled by different sets of genes, therefore mutational changes can easily be restricted to one or two types of organ by mutations in the genes that specify their identity during development. For example, mutational changes can be easily restricted to the inner tepals and lip by mutations in a clade 3 gene, or to the lip by mutations in a clade 4 gene. This may provide a proximate explanation for why the lip is the most diverse organ. A similar scenario is not likely in petaloid monocots with identical tepals, because changes in the mechanisms determining organ identity of one tepal are very likely accompanied by the same (pleiotropic) changes in the rest of the perianth due to their common genetic control of development [62].

The 'orchid code' suggests that the diversification of the orchid perianth started with changes in the regulatory regions of the duplicated class B genes, which were soon followed by changes that led to the recognition of different target genes [42]. The conserved clade-specific motifs that characterize each clade may be the result of diversifying selection taking place *early *and *transiently *during the divergence of the ancestral paralogues, but possibly after some changes that affected the domains of expression had already occurred. Subsequently, purifying selection may have been stabilizing the network of protein – protein and protein – DNA interactions involving four DEF-like proteins.

Our analyses suggest that diversification of the K-domain in *DEF*-like genes of Poales may have triggered the initial changes in transcription factor interactions that coordinate the development of the lodicules (Figure 6). Our comparative analysis of class B genes in the Orchidaceae and Poales, as well as published information on their pattern of expression, suggests that in these species-rich groups positive selection and transcriptional divergence have had different influences on the evolution of morphological diversification. In a wider context, the presented results suggest the preservation and functional diversification of paralogous genes involves case-specific differential divergence of coding and regulatory sequences (Figure 6).

## Methods

### Plant material

Pre-anthesis flower buds of *Hypoxis villosa *(Hypoxidaceae) and orchids *Vanilla planifolia *(Vanilloideae),*Phragmipedium longifolium *(Cypripedioideae), *Spiranthes odorata *(Orchidoideae) and *Gongora galeata *(Epidendroideae) were collected from the living collections of the Halle (Halle an der Saale), Wilhelma (Stuttgart) and Heidelberg Botanical Gardens. They were preserved and transported in liquid nitrogen or RNAlater (Sigma) and stored at -80°C. The choice of the orchid species was based on their subfamily membership, the availability of blooming individuals and the number of plants present in living collections. In this study, we did not include material from the subfamily Apostasioideae because there were no specimens available in the living orchid collections outside of their natural range of distribution in Southeast Asia. We included *H. villosa *because this species and the rest of the family Hypoxidaceae (Asparagales) show important similarities to the orchids [98,99] and members of the Hypoxidaceae are frequently used as point of comparison in recent molecular phylogenies of the orchids.

### Sequence isolation

Bud material was used for total RNA isolation with Biomol's reagent, following the protocol of the manufacturer. Total RNA was employed for cDNA synthesis by using a poly-T primer with Fermentas MuLV Reverse Transcriptase. MADS-box gene specific sequences were isolated by 3' RACE from at least two different cDNA pools from each species with primer pair RQVT2 (5'-CGR CAR GTG ACS TTC TSC AAR CG-3') and AB07 (5'-GAC TCG AGT CGA CAT CTG-3') under conditions specified by the manufacturer for *Taq *polymerase (Fermentas). PCR products of about 700 bp were cloned into vectors pGEMT (Promega) or pJET1 (Fermentas) following the protocols of the manufacturers. The ligation products where electroporated into *E. coli *XL1 Blue, and 50 to 100 positive clones from each ligation were selected and sequenced in both directions multiple times with vector-specific primers on an ABI 3730xl DNA Analyzer using Big Dye Terminator chemistry. The resulting sequences were assembled and managed with the program SEQUENCHER (4.5, Gene Codes Corporation). The level of identity and phylogenetic association of these sequences with previously identified monocot *DEF*- and *GLO*-like sequences was determined with different strategies using BLAST [100] to all plant sequences in GenBank. The sequences that where unequivocally identified as those of *DEF- *or *GLO*-like transcripts were employed to generate specific primers to isolate the 5' end of the sequence with the 5'-RACE kit (Roche). Assembly of the 5' and 3' partial sequences allowed us to design specific primers to isolate the corresponding full-length sequences.

### Sequence alignment

We assembled two datasets by retrieving with BLAST and keyword searches all monocot *DEF*- or *GLO*-like nucleotide and amino acid sequences deposited in NCBI's GenBank until September 20^th ^2007 (Table S1 in Additional file 1). The amino acid sequences were aligned using the program T-coffee [101,102] to the corresponding DEF- and GLO-like conceptual amino acid translations of the cDNA sequences that we isolated from orchids. In these alignments we included as outgroup representatives the following sequences from basal angiosperms: *KjAP3 *(*Kadsura japonica*),*AP3-1 *(*Illicium floridanum*),*IhAP3-1 *(*Illicium henryi*),*BsAP3 *(*Brasenia schreberi*),*NjAP3-3 *(*Nuphar japonica*),*EfAP3 *(*Euryale ferox*),*NymAP3 *(*Nymphaea sp*.), *NtAP3_1 *(*Nymphaea tetragona*),*AP3-1 *(*Nuphar variegata*) and *AmAP3 *(*Amborella trichopoda*) for the phylogeny of *DEF*-like sequences. Similarly, for the phylogeny of *GLO*-like genes we employed the sequences of *KjPI *(*Kadsura japonica*), *PI *(*Illicium floridanum*), *IhPI-1 *(*Illicium henryi*), *BsPI *(*Brasenia schreberi*), *CcPI *(*Cabomba caroliniana*), *NtPI *(*Nuphar tetragona*), *EfPI *(*Euryale ferox*), *NjPI-1*, *NjPI-2 *(*Nuphar japonica*) and *AtPI *(*Amborella trichopoda*). In an initial step, the alignments were assessed with GBLOCKS [103] and T-coffee. Subsequently, after manual improvement using GenDoc [104], we used amino-acid sequence alignments as a template to align the corresponding nucleotide sequences. The nucleotide alignment of *DEF*-like genes contained 61 sequences and was 762 bp long, whereas the *GLO*-like matrix contained 74 sequences and was 696 bp long.

### Phylogenetic reconstruction and analysis

We analyzed the alignments of *DEF*- and *GLO*-like sequences on their complete length or separated in the positions encoding the MIK region and the C-terminal domain, as defined in [71]. The program MODELTEST (3.7) [105] was employed to determine which model of nucleotide substitution fits better to each alignment according to the corrected Akaike Information Criterion [106]. The parameters of the best model were employed to reconstruct the phylogenies of *DEF*- and *GLO*-like genes with Mr. Bayes (3.1.2) [107]. We performed preliminary runs of Bayesian Inference to determine the point where the Likelihood estimates resulting from each generation converges on a single value. Based on this, we performed all analyses of Bayesian inference for 2,000,000 generations, sampling every 100th and with a burn-in of 3,000 generations. Finally, we obtained a consensus tree with the rest of the results and used the posterior probabilities (PP) to estimate the statistical support for each node.

### Determining the substitution saturation of the dataset

We determined the level of substitution saturation of *DEF*- and *GLO*-like sequences by plotting their genetic distance versus the number of transitions and transversions. Because the number of transitions relative to that of transversions decreases with increasing divergence, the point of substitution saturation corresponds with the distance value where the plots diverge from linearity and reach a plateau [108]. The pairwise genetic distances of both *DEF*- and *GLO*-like genes were obtained in PAUP 4 beta 10 (Swofford 2002) using the nucleotide substitution model GTR+I+G which best fitted the data as estimated by analysis with MODELTEST (see previous section). The pairwise number of transitions and transversions corresponding to each dataset was obtained using DAMBE 4.5.56 [108] and the plots were drawn with MicroSoft Excel.

### Relative Rate Test

We employed the Maximum Likelihood (ML) pairwise Relative Rate Test (RRT) as implemented in the program HyPhy (v. 1.0) [109] to estimate the relative rate of substitution between the orchid and Poales *DEF*- and *GLO*-like sequences and their corresponding outgroup sequence *TGDEFA *and *TGGLO *from *Tulipa gesneriana *(Liliaceae), respectively. We chose these as representatives of the outgroup because the phylogenetic analysis presented in this paper showed that their relationship to the sequences from the rest of the monocots is well supported and their level of nucleotide substitution is not saturated.

The Maximum Likelihood-based RRT in HyPhy employs a Likelihood Ratio Test (LRT) to evaluate whether the sequence data fits the null hypothesis of a molecular clock (equal rates) represented by a tree of three taxa where all parameters are constrained to be equal along its branches, or an alternative hypothesis where such parameters are free to adopt different values. In order to infer the parameters of these phylogenetic hypotheses for each pair of sequences, we used the Muse-Gaut model [110] of codon substitution with nucleotide equilibrium frequencies based on their position in the codon (in Hyphy nomenclature MG94W9). The resulting parameter estimates were compared through series of Likelihood Ratio Tests that involved all pairs of sequences in relation to the corresponding outgroups. There is no simple procedure to reduce the effect of multiple comparisons in this series of *P*-values because the RRTs share the same outgroup and thus are not independent. Alternatively, the developers of HyPhy recommend adjusting the *P*-values of the LRTs for False Discovery Rate by implementing the Benjamini-Hochberg procedure (S. Kosakovsky-Pond, Hyphy on-line discussion forum). The False Discovery Rate (FDR) is the proportion of false positive results among all significant results. With the Benjamini-Hochberg correction we enforced a FDR = 0.05 by ranking all n *P*-values from smallest to largest, then dividing them by n and multiplying the result by 0.05 [111]. The LRT comparisons that rejected the null hypothesis of neutrality are those where the original *P*-value was smaller than the corrected value.

For consistency with analyses described in the following section, the RRT analysis excludes sequences from basal angiosperms and all positions with indels.

### Analysis of evolutionary patterns of divergence

The ratio (*ω*) of the rate of nonsynonymous substitutions at nonsynonymous sites (dN) to synonymous (dS) substitutions at synonymous sites was estimated to figure out whether the coding region of a gene is under negative (purifying) selection (*ω *< 1), positive selection (*ω *> 1) or evolves neutrally (*ω *= 1). Variations of *ω *along the evolutionary history of a gene family indicate changes in both the associated selective regime and functional constraints. Because different selective regimes might affect only some branches or sites during relatively short evolutionary time [6], we analyzed the heterogeneity of selective pressures in *DEF*- and *GLO*-like genes with the program codeml from the PAML package [112,113], by comparing five pairs of models (abbreviated M) that describe the pattern of codon substitution at the levels of: (a) codon sites (M7 vs M8), (b) specific branches in a phylogeny (M0 vs M2) and (c) sites from specific clades (M1a vs MC and M3 vs MD) or (d) sites from specific branches (MA and MA1). Table 1 summarizes the main features and parameters of each model of codon substitution and the datasets analyzed with them. Each of these models serves to estimate *ω *and other parameters. Comparing the likelihood of these estimates via a Likelihood Ratio Test (LRT) determines whether a model that considers positive selection fits the data better than one assuming neutral or purifying selection. A detailed description of each test is provided elsewhere [70,112,114].

We investigated the occurrence of positive selection along codon sites by comparing the model M8 with model M7 [112]. Then, we characterized the variation in selection pressure among branches of *DEF*- and *GLO*-like genes with a series of tests comparing the null hypothesis M0 "one *ω *ratio for all branches", with different alternative hypothesis based on M2 "different *ω *ratios", where independent *ω *values are estimated for specific branches [115] (Table 1).

We implemented clade and site tests of models M1a vs MC and M3 vs MD, to determine whether functional constraints differ significantly between clades after gene duplication in the two paralogous groups of *DEF*-like genes in Orchidaceae (Clades 1 and 2) or (Clades 3 and 4) (Figure 1), or the duplicate *GLO*-like genes from Poales (Figure 2). Clade models MC and MD assume that there were variations in the selective pressures among the amino acids encoded by a gene and that some of these sites also experienced changes in selective regimes at different points of their evolutionary history, such as changes occurring after gene duplication [70] (Table 1).

With branch-site models A and A1 [116], we tested for the occurrence of positive selection on individual codons along specific branch groups of *DEF*- and *GLO*-like genes from the orchids and Poales. Model A assumes that the branches in the phylogeny are divided *a priori *in foreground and background clades, where only the former may have experienced positive selection (Table 1). In the null model MA1 *ω*_2 _= 1, thus comparing model MA with MA1 is a direct test of positive selection on the foreground clades.

The relative fit of the parameters estimated by each of these models of codon substitution is represented by a maximum likelihood value that can be compared between nested hypotheses by a Likelihood Ratio Test (LRT). The LRT statistics are assumed to be χ^2 ^distributed with degrees of freedom equal to the difference in the number of parameters between models. The application of these models requires a phylogenetic assumption, but since the process of ML inference with program codeml is computationally intensive, for most of the tests we employed unrooted trees inferred as previously described from a smaller dataset. This dataset does not include sequences of basal angiosperms and sequences with large sections of indels (gaps) because we did not want to consider them as additional character states. The alignment of *DEF*-like genes is 747 bp long and contains 41 sequences, while the alignment of *GLO*-like genes is 666 bp long and has 61 sequences. The tests involving clade-sites models required the use of three alignments that only included members of clades 1 and 2, 3 and 4 from the Orchidaceae *DEF*-like genes or sequences from clades 1 and 2 of the Poales, respectively (Table 1). The phylogenies corresponding to these alignments were constructed as described previously.

To detect variations in parameter estimation, we performed and compared the analyses of each model at least twice. As with the RRTs with HyPhy, all analyses with codeml were conducted without considering positions with indels. In addition to the tests performed with PAML, we carried out an independent estimation of ω in *DEF*-like genes with the on-line implementation of the Hyphy package . Specifically, we employed the FEL (Fixed Effects Likelihood) method to estimate the rates of nonsynonymous and synonymous substitution across codons with the same alignment and phylogeny previously used to test M0 with PAML.

## Authors' contributions

MMP and GT designed and coordinated study; MMP collected material; MK contributed with plant material and laboratory space; MMP, LH and AH performed RACE, cloning and sequencing of the *DEF *and *GLO*-like genes from orchids and *Hypoxis *newly reported in this study. MMP performed phylogenetic and molecular evolution analyses; MMP drafted the manuscript; MMP, GT and MK edited the manuscript. All authors read and approved the final manuscript.

## Supplementary Material

Additional file 1 **Supplemental tables**. Table S1. *DEF- *and *GLO*-like sequences employed in this study. This manuscript describes the isolation and characterization of those sequences with names in bold type. Table S2. Parameter estimates and LRT of M7 and M8 from monocots. Table S3. Parameter estimates and LRT of MA1 vs. MA in *DEF*-like genes from Orchidaceae. The branches tested are those labeled with cursive fonts in Figure 3. Table S4. Parameter estimates and LRT of MA1 vs. MA in *GLO*-like genes from Orchidaceae and Poales. The branches tested are those labeled with fonts in italics in Figure 4. Click here for file

Additional file 2 **Condensed alignment of DEF-like proteins from Asparagales included in the phylogenetic analysis illustrated by Figure **1** and Additional files **4** and **5. This alignment only includes variable amino-acid positions shaded according to their chemical property: Red background/green fonts = D, E; Red background/white fonts = H, K, R; Yellow background/green fonts = N, Q; Yellow background/black fonts = S, T; Bright green background/red fonts = L, I, V; Bright green background/white fonts = F, Y, W; Dark green background/red fonts = A, G; Dark green background/white fonts = M, C; White background/black fonts = P. Click here for file

Additional file 3 **Condensed alignment of GLO-like proteins from Asparagales included in the phylogenetic analysis illustrated by Figure **2** and Additional files **6**and **7. This alignment only includes variable amino-acid positions shaded according to their chemical property as described for Supplementary Figure 1. Click here for file

Additional file 4 **Phylogeny of monocot *DEF*-like genes based on the C-terminal domain.** Click here for file

Additional file 5 **Phylogeny of monocot *DEF*-like genes based on the regions encoding the MIKC-domains.** Click here for file

Additional file 6 **Phylogeny of monocot *GLO*-like genes based on the positions encoding the MIK-domains.** Click here for file

Additional file 7 **Phylogeny of monocot *GLO*-like genes based on the positions encoding the C- terminal domain.** Click here for file

Additional file 8 **Relative rates of A) nonsynonymous and B) synonymous substitution in *DEF*-like sequences from the Orchidaceae**. Each bar represents the median of the corresponding relative rate of substitution between the pairs of two groups of sequences that yielded statistically significant results after correction for multiple comparisons. All data represented here are the result of two or more comparisons and exclusively involves orchid genes. Click here for file
