# Supplementary material for: Positive selection and ancient duplications in the evolution of class B floral homeotic genes of orchids and grasses
Source: BMC Evol Biol. 2009 Apr 21;9:81. doi: 10.1186/1471-2148-9-81 (PMC2680841; doi:10.1186/1471-2148-9-81)
Supplement: Additional file 1 — Supplemental tables. Table S1. DEF- and GLO-like sequences employed in this study. This manuscript describes the isolation and characterization of those sequences with names in bold type. Table S2. Parameter estimates and LRT of M7 and M8 from monocots. Table S3. Parameter estimates and LRT of MA1 vs. MA in DEF-like genes from Orchidaceae. The branches tested are those labeled with cursive fonts in Figure 3. Table S4. Parameter estimates and LRT of MA1 vs. MA in GLO-like genes from Orchidaceae and Poales. The branches tested are those labeled with fonts in italics in Figure 4. [file 1471-2148-9-81-S1.pdf]

Table S1. *DEF* and *GLO*-like sequences employed in this study. This manuscript describes the isolation and characterization of those sequences with names in bold type.

***DEF*-like sequences**	**Species**	**GenBank Accession Number**	**Order**	***GLO*-like sequences**	**Species**	**GenBank Accession Number**	**Order**
AmAP3	*Amborella trichopoda*	AB154845	Amborellales	*PI*	*Acorus calamus*	DQ005577	Acorales
DEF1	*Elaeis guineensis*	AY739700	Arecales	*PI*	*Alisma plantago-aquatica*	DQ005581	Alismatales
ApDEF	*Agapanthus praecox*	AB177941	Asparagales	*PI*	*Arum maculatum*	DQ005580	Alismatales
AODEF	*Asparagus officinalis*	AB094964	Asparagales	*SmPI*	*Sagittaria montevidensis*	AF230712	Alismatales
MADS1	*Cymbidium spp*	DQ683575	Asparagales	*PIa*	*Tofieldia calyculata*	DQ005578	Alismatales
DcOAP3A	*Dendrobium crumenatum*	DQ119838	Asparagales	*PIb*	*Tofieldia calyculata*	DQ005579	Alismatales
DcOAP3B	*Dendrobium crumenatum*	DQ119839	Asparagales	*AmPI*	*Amborella trichopoda*	AB154841	Amborellales
GogalDEF1	***Gongora galeata***	FJ804097	Asparagales	*PI1*	*Caryota mitis*	DQ005601	Arecales
GogalDEF2	***Gongora galeata***	FJ804098	Asparagales	*PI2*	*Caryota mitis*	DQ005600	Arecales
GogalDEF3	***Gongora galeata***	FJ804099	Asparagales	*GLO1*	*Elaeis guineensis*	AF227195	Arecales
HrDEF	*Habenaria radiata*	AB232663	Asparagales	*GLO2*	*Elaeis guineensis*	AF411848	Arecales
MADS1	*Hemerocallis hyb*	AF209729	Asparagales	*ApMADS1*	*Agapanthus praecox*	AB079259	Asparagales
HyvilDEF1	***Hypoxis villosa***	FJ804101	Asparagales	*PI2*	*Allium ursinum*	DQ005594	Asparagales
HyvilDEF2	***Hypoxis villosa***	FJ804102	Asparagales	*AOGLOA*	*Asparagus officinalis*	AB103465	Asparagales
MaDEF	*Muscari armeniacum*	AB201751	Asparagales	*AOGLOB*	*Asparagus officinalis*	AB103466	Asparagales
OMADS3	*Oncidium* "Gower Ramsey"	AY196350	Asparagales	*PIC2*	*Crocus sativus*	DQ231251	Asparagales
PeMADS2	*Phalaenopsis equestris*	AY378149	Asparagales	*DthyrPI*	*Dendrobium thyrsiflorum*	DQ017701	Asparagales
PeMADS3	*Phalaenopsis equestris*	AY378150	Asparagales	*PI*	*Epipactis palustris*	DQ005588	Asparagales
PeMADS4	*Phalaenopsis equestris*	AY378147	Asparagales	***GogalGLO1***	***Gongora galeata***	FJ804100	Asparagales
PeMADS5	*Phalaenopsis equestris*	AY378148	Asparagales	*HrGLO1*	*Habenaria radiata*	AB232665	Asparagales
PhlonDEF1	***Phragmipedium longifolium***	FJ804105	Asparagales	*HrGLO2*	*Habenaria radiata*	AB232664	Asparagales
PhlonDEF2	***Phragmipedium longifolium***	FJ804106	Asparagales	***HyvilGLO1***	***Hypoxis villosa***	FJ804103	Asparagales
PhlonDEF3	***Phragmipedium longifolium***	FJ804107	Asparagales	***HyvilGLO2***	***Hypoxis villosa***	FJ804104	Asparagales
PhlonDEF4	***Phragmipedium longifolium***	FJ804108	Asparagales	*PI1*	*Muscari botryoides*	DQ005597	Asparagales
SpodoDEF1	***Spiranthes odorata***	FJ804110	Asparagales	*PI2*	*Muscari botryoides*	DQ005596	Asparagales
SpodoDEF2	***Spiranthes odorata***	FJ804111	Asparagales	*PI1a*	*Narcissus cyclamineus*	DQ005599	Asparagales
SpodoDEF3	***Spiranthes odorata***	FJ804112	Asparagales	*PI1b*	*Narcissus cyclamineus*	DQ005599	Asparagales
SpodoDEF4	***Spiranthes odorata***	FJ804113	Asparagales	*OrcPI*	*Orchis italica*	AB094985	Asparagales
VaplaDEF1	***Vanilla planifolia***	FJ804115	Asparagales	*PeMADS6*	*Phalaenopsis equestris*	AY678299	Asparagales
VaplaDEF2	***Vanilla planifolia***	FJ804116	Asparagales	***PhlonGLO1***	***Phragmipedium longofolium***	FJ804109	Asparagales
VaplaDEF3	***Vanilla planifolia***	FJ804117	Asparagales	*PI1a*	*Speirantha convallarioides*	DQ005605	Asparagales
Ap3-1	*Illicium floridanum*	AY936225	Austrobaileyales	*PI1b*	*Speirantha convallarioides*	DQ005606	Asparagales
IhAP3-1	*Illicium henryi*	AY436729	Austrobaileyales	***SpodoGLO1***	***Spiranthes odorata***	FJ804114	Asparagales
KjAP3	*Kadsura japonica*	AB154848	Austrobaileyales	***VaplaGLO1***	***Vanilla planifolia***	FJ804118	Asparagales
CCDEF	*Commelina communis*	AB177808	Commelinales	*PI*	*Illicium floridanum*	AY936224	Austrobaileyales

TRDEF	*Tradescantia reflexa*	AB177806	Commelinales
AP3	*Tacca chantrieri*	AF230706	Dioscoreales
AlsDEFa	*Alstromeria ligtu*	AB267842	Liliales
AlsDEFb	*Alstromeria ligtu*	AB267843	Liliales
MADS1	*Lilium longiflorum*	AF503913	Liliales
LRDEF1	*Lilium regale*	AB071378	Liliales
TriaDEF	*Tricyrtis affinis*	AB244024	Liliales
TGDEFA	*Tulipa gesneriana*	AB094965	Liliales
TGDEFB	*Tulipa gesneriana*	AB094966	Liliales
MagrAP3	*Magnolia grandiflora*	AY337752	Magnoliales
BsAP3	*Brasenia schreberi*	AB158355	Nympheales
EfAP3	*Euryale ferox*	AB158349	Nympheales
NjAP3-2	*Nuphar japonica*	AB158358	Nympheales
AP3-1	*Nuphar variegata*	AY337745	Nympheales
NymAP3	*Nymphaea tetragona*	AB158351	Nympheales
AP3	*Elegia elephas*	DQ662239	Poales
AP3b	*Elegia elephas*	DQ662240	Poales
HvAP3	*Hordeum vulgare*	AY541065	Poales
AP3	*Joinvillea ascendens*	DQ662238	Poales
SPW1	*Oryza sativa*	NM_001065095	Poales
AP3	*Streptochaeta angustifolia*	DQ662237	Poales
TaMADS51	*Triticum aestivum*	AB007506	Poales
TaMADS82	*Triticum aestivum*	AB107993	Poales
SILKY1	*Zea mays*	AF181479	Poales
MADS5	*Alpinia hainanensis*	AY621154	Zingiberales

IhPI-1	*Illicium henryi*	AY436734	Austrobaileyales
KjPI	*Kadsura japonica*	AB154849	Austrobaileyales
CCGLO	*Commelina communis*	AB177807	Commelinales
TcPI	*Tacca chantrieri*	AF230713	Dioscoreales
PI	*Alstromeria ligtu*	DQ005593	Liliales
GLO1	*Lilium longiflorum*	DQ437527	Liliales
PI1	*Lilium martagon*	DQ005592	Liliales
LRGLOA	*Lilium regale*	AB071379	Liliales
TriaGLO	*Tricyrtis affinis*	AB244023	Liliales
TGGLO	*Tulipa gesneriana*	AB094967	Liliales
PIa	*Veratrum californicum*	DQ005589	Liliales
PIb	*Veratrum californicum*	DQ005590	Liliales
BsPI	*Brasenia schreberi*	AB158356	Nympheales
CcPI	*Cabomba caroliniana*	AB158354	Nympheales
EfPI	*Euryale ferox*	AB158350	Nympheales
NjPI-1	*Nuphar japonica*	AB158359	Nympheales
NjPI-2	*Nuphar japonica*	AB158360	Nympheales
NtPI	*Nymphaea tetragona*	AB158352	Nympheales
PI1	*Cortaderia selloana*	DQ005583	Poales
PI2	*Cortaderia selloana*	DQ005584	Poales
PI	*Elegia elephas*	DQ662246	Poales
HvPI	*Hordeum vulgare*	AY541066	Poales
PI	*Joinvillea ascendens*	DQ662245	Poales
MADS2	*Oryza sativa*	AY551924	Poales
MADS4	*Oryza sativa*	L37527	Poales
PI1	*Pharus virescens*	DQ662243	Poales
PI2	*Pharus virescens*	DQ662242	Poales
PI1	*Streptochaeta angustifolia*	DQ662244	Poales
PI2	*Streptochaeta angustifolia*	DQ662241	Poales
WPI1	*Triticum aestivum*	AB107991	Poales
WPI2	*Triticum aestivum*	AB107992	Poales
ZMM16	*Zea mays*	NM_001111666	Poales
ZMM18	*Zea mays*	AJ292960	Poales
ZMM29	*Zea mays*	NM_001111667	Poales
MADS8	*Alpinia hainanensis*	AY621156	Zingiberales
PI	*Alpinia oblongifolia*	DQ286723	Zingiberales
PI	*Globba marantina*	DQ005602	Zingiberales
MADS1	*Musa acuminata*	AY941798	Zingiberales
PIa	*Musa ornata*	DQ005604	Zingiberales
PIb	*Musa ornata*	DQ005603	Zingiberales

Table S2. Parameter estimates and LRTs analyses with models M7 vs. M8 from monocots.

**Model**	**Estimate of parameters**	**L**
DEF-like		
M7 (beta)	p= 0.53871 q= 4.71490	-9756.581685
M8 (beta and ω)	p_0= 1.00000 p= 0.54102 q= 4.76098 (p_1= 0.00000) ω= 2.47868	-9756.521674
LRT<br>M7 vs M8 : 2δ =0.12002 df=2 P=0.729		
GLO-like		
Model	Estimate of parameters	L
M7 (beta)	p=0.80996 q=5.13571	-11756.678564
M8 (beta and ω)	p_0=1.00000 p=0.81307 q= 5.17033 (p_1=0.00000) ω= 3.17976	-11756.551266
LRT<br>M7 vs M8 : 2δ =0.254596 df=2 P=0.8805		

Table S3. Parameter estimates and LRT of MA1 vs. MA in *DEF*-like genes from Orchidaceae. The branches tested are those labeled with cursive fonts in Figure 3.

Clade	Model	Estimate of parameters	Positive selection	L
C1<br>(*O1*)	A	ω_0=0.08415, f_0=0.96395<br>ω_1=1.00000, f_1=0.03605<br>$\omega_{2a\ fore}$ =1.00000, $\omega_{2a\ back}$ =0.08415, f_{2a} =0<br>$\omega_{2b\ fore}$ =1.00000, $\omega_{2b\ back}$ =1.00000, f_{2b} =0	73 Q 0.752, 114 L 0.546, 125 N 0.608, 131 K 0.516	-9988.457096
	A1	ω_0=0.08415, f_0=0.96395<br>ω_1=1.00000, f_1=0.03605<br>$\omega_{2a\ fore}$ =1.00000, $\omega_{2a\ back}$ =0.08415, f_{2a} =0<br>$\omega_{2b\ fore}$ =1.00000, $\omega_{2b\ back}$ =1.00000, f_{2b} =0		-9988.457096
LRT	2δ = 0 df=1 P=1			
C2<br>(*O2*)	A	ω_0=0.08350, f_0=0.00015<br>ω_1=1.00000, f_1=0.00001<br>$\omega_{2a\ fore}$ =3.14115, $\omega_{2a\ back}$ =0.08350, f_{2a} =0.96354<br>$\omega_{2b\ fore}$ =3.14115, $\omega_{2b\ back}$ =1.00000, f_{2b} =0.03630	16 N 0.966*, 51 T 0.982*, 69 D 0.511, 79 L 0.980*, 130 L 0.615, 170 N 0.588, 171 Y 0.972*	-9987.380581
	A1	ω_0=0.08354, f_0=0.00001<br>ω_1=1.00000, f_1=0.00000<br>$\omega_{2a\ fore}$ =1.00000, $\omega_{2a\ back}$ =0.08354, f_{2a} =0.96365<br>$\omega_{2b\ fore}$ =1.00000, $\omega_{2b\ back}$ =1.00000, f_{2b} =0.03635		-9987.388991
LRT	2δ =0.01682 df=1 P=0.8963			
C3<br>(*O3*)	A	ω_0=0.08415, f_0=0.96395<br>ω_1=1.00000, f_1=0.03605<br>$\omega_{2a\ fore}$ =1.00000, $\omega_{2a\ back}$ =0.08415, f_{2a} =0.00000<br>$\omega_{2b\ fore}$ =1.00000, $\omega_{2b\ back}$ =1.00000, f_{2b} =0.00000	33 T 0.505, 43 V 0.628, 68 F 0.641, 178 D 0.539	-9988.457096
	A1	ω_0=0.08415, f_0=0.96395<br>ω_1=1.00000, f_1=0.03605<br>$\omega_{2a\ fore}$ =1.00000, $\omega_{2a\ back}$ =0.08415, f_{2a} =0.00000<br>$\omega_{2b\ fore}$ =1.00000, $\omega_{2b\ back}$ =1.00000, f_{2b} =0.00000		-9988.457096
LRT	2δ =0 df=1 P=1			
C4<br>(*O4*)	A	ω_0=0.08415, f_0=0.96395<br>ω_1=1.00000, f_1=0.03605<br>$\omega_{2a\ fore}$ =1.00000, $\omega_{2a\ back}$ =0.08415, f_{2a} =0.00000<br>$\omega_{2b\ fore}$ =1.00000, $\omega_{2b\ back}$ =1.00000, f_{2b} =0.00000		-9988.457096
	A1	ω_0=0.08415, f_0=0.96395<br>ω_1=1.00000, f_1=0.03605<br>$\omega_{2a\ fore}$ =1.00000, $\omega_{2a\ back}$ =0.08415, f_{2a} =0<br>$\omega_{2b\ fore}$ =1.00000, $\omega_{2b\ back}$ =1.00000, f_{2b} =0		-9988.457096
LRT	2δ =0 df=1 P=1			
C1-C2<br>(*O5*)	A	ω_0=0.08415, f_0=0.96395<br>ω_1=1.00000, f_1=0.03605<br>$\omega_{2a\ fore}$ =1.00000, $\omega_{2a\ back}$ =0.08415, f_{2a} =0.00000<br>$\omega_{2b\ fore}$ =1.00000, $\omega_{2b\ back}$ =1.00000, f_{2b} =0.00000	99 R 0.694	-9988.457096
	A1	ω_0=0.08400, f_0=0.95367<br>ω_1=1.00000, f_1=0.03556<br>$\omega_{2a\ fore}$ =1.00000, $\omega_{2a\ back}$ =0.08400, f_{2a} =0.01039<br>$\omega_{2b\ fore}$ =1.00000, $\omega_{2b\ back}$ =1.00000, f_{2b} =0.00039		-9988.388303
LRT	2δ = -0.137586 df=1 P=1			
C3-C4<br>(*O6*)	A	ω_0=0.08415, f_0= 0.96395<br>ω_1=1.00000, f_1=0.03605<br>$\omega_{2a\ fore}$ =1.00000, $\omega_{2a\ back}$ =0.08415, f_{2a} =0.00000<br>$\omega_{2b\ fore}$ =1.00000, $\omega_{2b\ back}$ =1.00000, f_{2b} =0.00000		-9988.457096
	A1	ω_0=0.08415, f_0=0.96395<br>ω_1=1.00000, f_1=0.03605<br>$\omega_{2a\ fore}$ =1.00000, $\omega_{2a\ back}$ =0.08415, f_{2a} =0.00000<br>$\omega_{2b\ fore}$ =1.00000, $\omega_{2b\ back}$ =1.00000, f_{2b} =0.00000		-9988.457096
LRT	2δ =0 df=1 P=1			

Table S4. Parameter estimates and LRT of MA1 vs. MA in *GLO*-like genes from Orchidaceae and Poales. The branches tested are those labeled with cursive fonts in Figure 4.

Clade	Model	Estimate of parameters	Positive selection	L
Poales (*P1*)	A	ω_0=0.11134, f_0=0.89830<br>ω_1=1.00000, f_1=0.04086<br>$\omega_{2a\ fore}$=1.69786, $\omega_{2a\ back}$=0.11134, f_{2a}=0.05820<br>$\omega_{2b\ fore}$=1.69786, $\omega_{2b\ back}$=1.00000, f_{2b}=0.00265	36 V 0.621<br>78 Q 0.673<br>128 Q 0.684	-12031.361375
	A1	ω_0=0.11125, f_0=0.87299<br>ω_1=1.00000, f_1=0.04002<br>$\omega_{2a\ fore}$=1.00000, $\omega_{2a\ back}$=0.11125, f_{2a}=0.08318<br>$\omega_{2b\ fore}$=1.00000, $\omega_{2b\ back}$=1.00000, f_{2b}=0.00381		-12031.456501
LRT	2δ=0.190252 df=1 P=0.6627			
Poales (*P2*)	A	ω_0=0.11235, f_0=0.84553<br>ω_1=1.00000, f_1=0.04310<br>$\omega_{2a\ fore}$=1.00000, $\omega_{2a\ back}$=0.11235, f_{2a}=0.10597<br>$\omega_{2b\ fore}$=1.00000, $\omega_{2b\ back}$=1.00000, f_{2b}=0.00540		-12032.453065
	A1	ω_0=0.11235, f_0=0.84553<br>ω_1=1.00000, f_1=0.04310<br>$\omega_{2a\ fore}$=1.00000, $\omega_{2a\ back}$=0.11235, f_{2a}=0.10597<br>$\omega_{2b\ fore}$=1.00000, $\omega_{2b\ back}$=1.00000, f_{2b}=0.00540		-12032.453065
LRT	2δ=0 df=1 P=1			
Poales (*P3*)	A	ω_0=0.11259, f_0=0.95192<br>ω_1=1.00000, f_1=0.04808<br>$\omega_{2a\ fore}$=1.00000, $\omega_{2a\ back}$=0.11259, f_{2a}=0.00000<br>$\omega_{2b\ fore}$=1.00000, $\omega_{2b\ back}$=1.00000, f_{2b}=0.00000		-12032.606000
	A1	ω_0=0.11259, f_0=0.95192<br>ω_1=1.00000, f_1=0.04808<br>$\omega_{2a\ fore}$=1.00000, $\omega_{2a\ back}$=0.11259, f_{2a}=0.00000<br>$\omega_{2b\ fore}$=1.00000, $\omega_{2b\ back}$=1.00000, f_{2b}=0.00000		-12032.606000
LRT	2δ=0 df=1 P=1			
Poales (*P4*)	A	ω_0=0.11222, f_0=0.79499<br>ω_1=1.00000, f_1=0.04038<br>$\omega_{2a\ fore}$=1.00000, $\omega_{2a\ back}$=0.11222, f_{2a}=0.15668<br>$\omega_{2b\ fore}$=1.00000, $\omega_{2b\ back}$=1.00000, f_{2b}=0.00796		-12032.198521
	A1	ω_0=0.11222, f_0=0.79499<br>ω_1=1.00000, f_1=0.04038<br>$\omega_{2a\ fore}$=1.00000, $\omega_{2a\ back}$=0.11222, f_{2a}=0.15668<br>$\omega_{2b\ fore}$=1.00000, $\omega_{2b\ back}$=1.00000, f_{2b}=0.00796		-12032.198521
LRT	2δ=0 df=1 P=1			
Orchids (*O1*)	A	ω_0=0.11259, f_0=0.95192<br>ω_1=1.00000, f_1=0.04808<br>$\omega_{2a\ fore}$=1.00000, $\omega_{2a\ back}$=0.11259, f_{2a}=0.00000<br>$\omega_{2b\ fore}$=1.00000, $\omega_{2b\ back}$=1.00000, f_{2b}=0.00000		-12032.606000
	A1	ω_0=0.11259, f_0=0.95192<br>ω_1=1.00000, f_1=0.04808<br>$\omega_{2a\ fore}$=1.00000, $\omega_{2a\ back}$=0.11259, f_{2a}=0.00000<br>$\omega_{2b\ fore}$=1.00000, $\omega_{2b\ back}$=1.00000, f_{2b}=0.00000		-12032.606000
LRT	2δ=0 df=1 P=1			
