# Supplementary material for: Positive selection and ancient duplications in the evolution of class B floral homeotic genes of orchids and grasses
Source: BMC Evol Biol. 2009 Apr 21;9:81. doi: 10.1186/1471-2148-9-81 (PMC2680841; doi:10.1186/1471-2148-9-81)
Supplement: Additional file 3 — Condensed alignment of GLO-like proteins from Asparagales included in the phylogenetic analysis illustrated by Figure 2 and Additional files 6and 7. This alignment only includes variable amino-acid positions shaded according to their chemical property as described for Supplementary Figure 1. [file 1471-2148-9-81-S3.pdf]

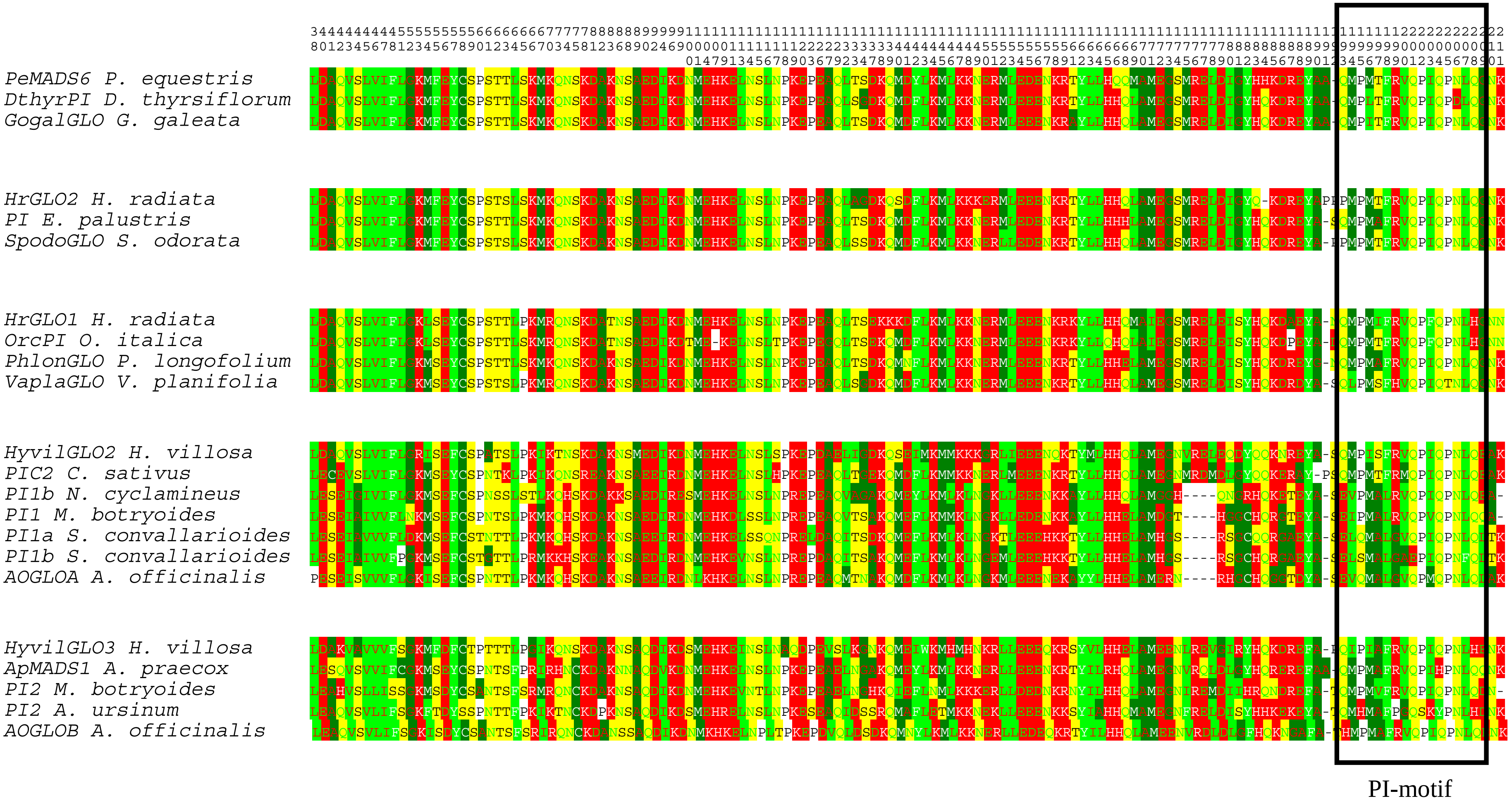
PeMADS6 P. equestris
DthyrPI D. thyrsiflorum
GogalGLO G. galeata
HrGLO2 H. radiata
PI E. palustris
SpodoGLO S. odorata
HrGLO1 H. radiata
OrcPI O. italica
PhlonGLO P. longofolium
VaplaGLO V. planifolia
HyvilGLO2 H. villosa
PIC2 C. sativus
PI1b N. cyclamineus
PI1 M. botryoides
PI1a S. convallarioides
PI1b S. convallarioides
AOGLOA A. officinalis
HyvilGLO3 H. villosa
ApMADS1 A. praecox
PI2 M. botryoides
PI2 A. ursinum
AOGLOB A. officinalis
PI-motif
