# Supplementary figures and images for: Positive selection and ancient duplications in the evolution of class B floral homeotic genes of orchids and grasses
Source: BMC Evol Biol. 2009 Apr 21;9:81. doi: 10.1186/1471-2148-9-81 (PMC2680841; doi:10.1186/1471-2148-9-81)

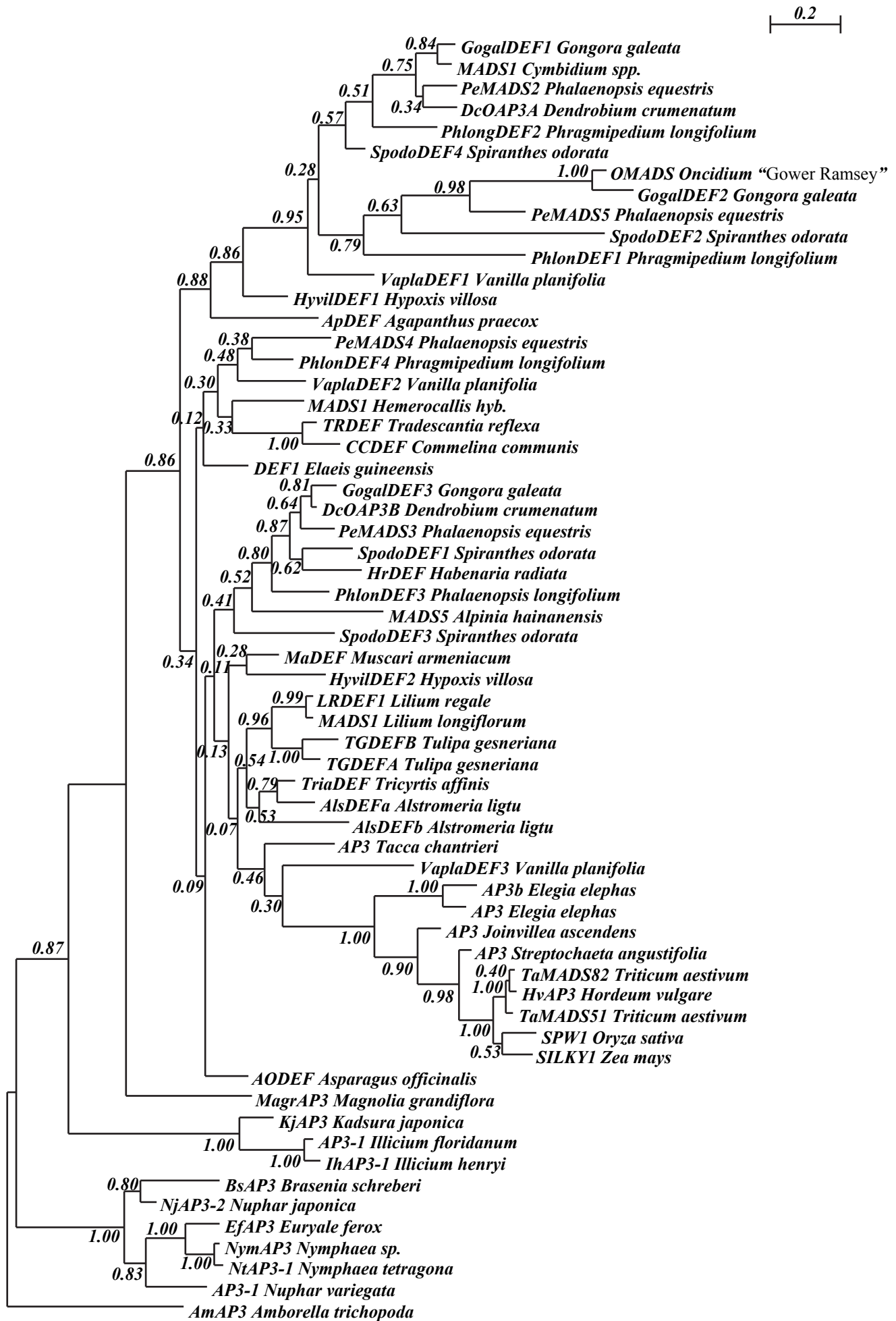

Supplement: Additional file 4 — Phylogeny of monocot DEF-like genes based on the C-terminal domain. [file 1471-2148-9-81-S4.pdf]

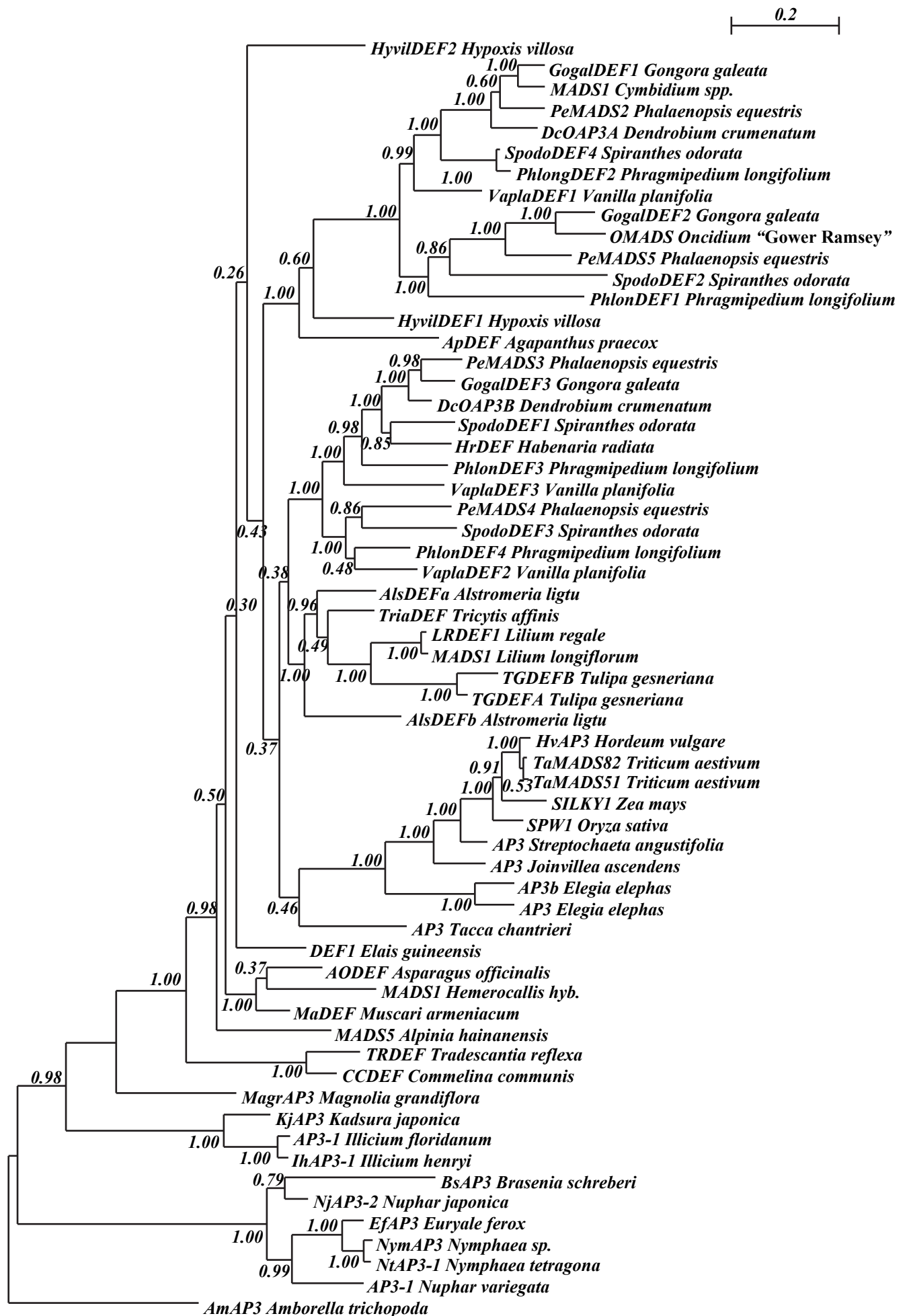

Supplement: Additional file 5 — Phylogeny of monocot DEF-like genes based on the regions encoding the MIKC-domains. [file 1471-2148-9-81-S5.pdf]

0.1

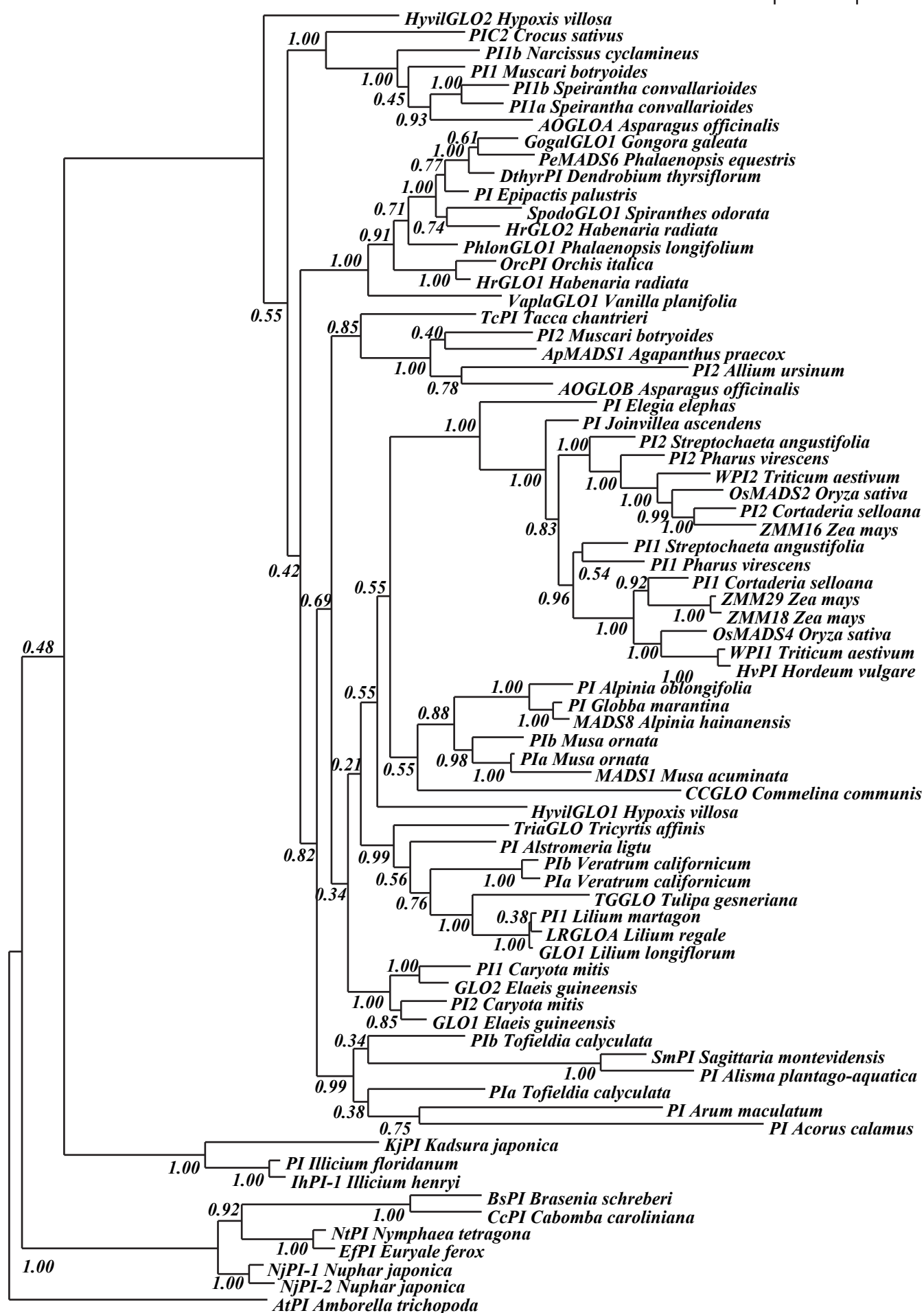

Supplement: Additional file 6 — Phylogeny of monocot GLO-like genes based on the positions encoding the MIK-domains. [file 1471-2148-9-81-S6.pdf]

0.1

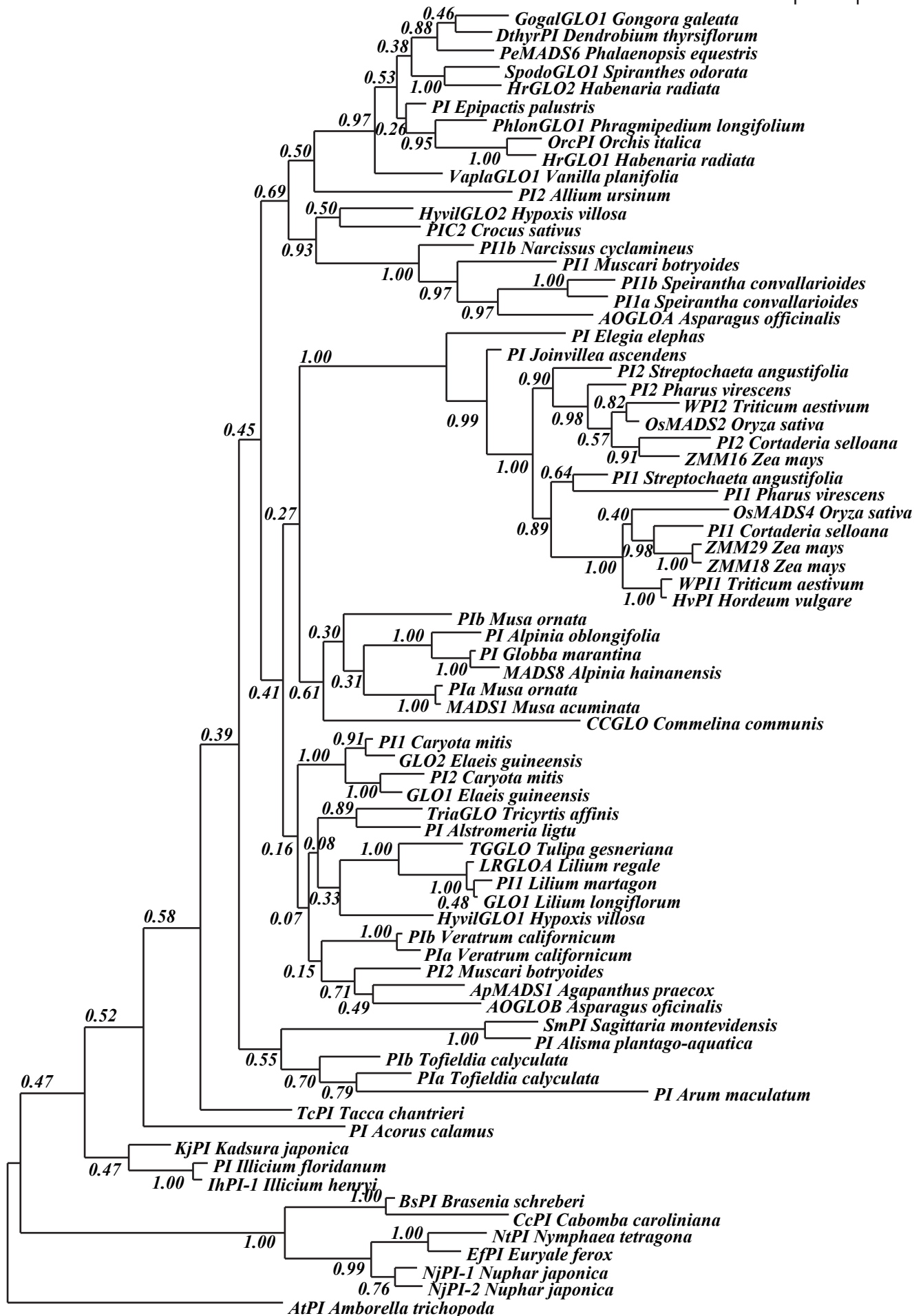

Supplement: Additional file 7 — Phylogeny of monocot GLO-like genes based on the positions encoding the C- terminal domain. [file 1471-2148-9-81-S7.pdf]

A)

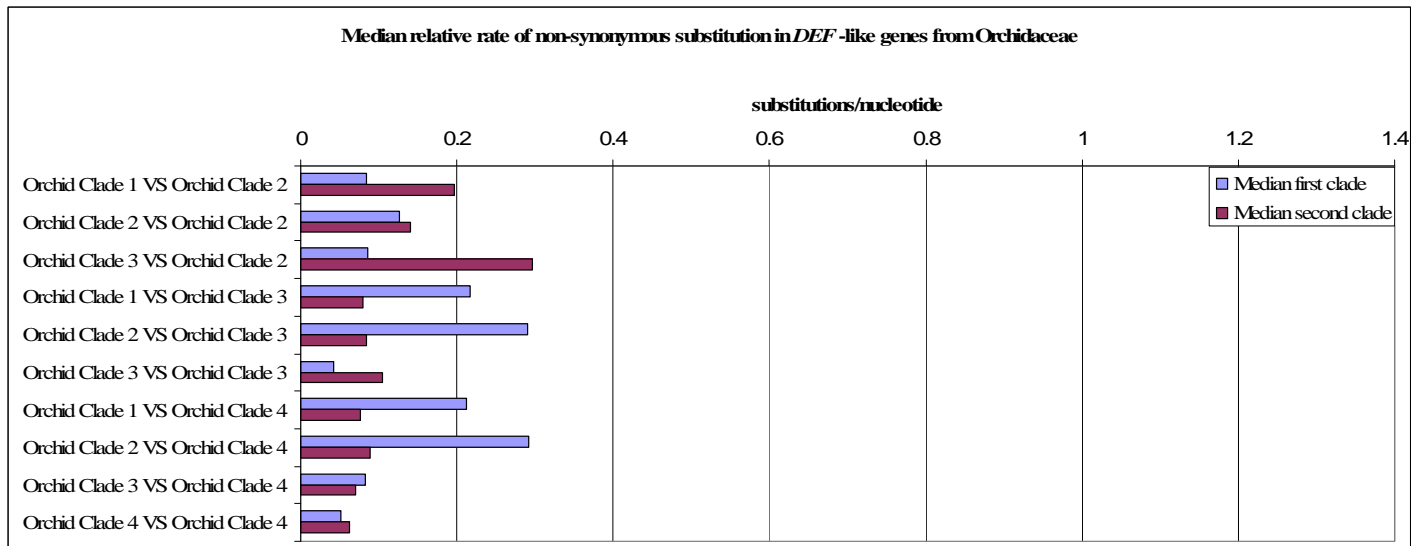

B)

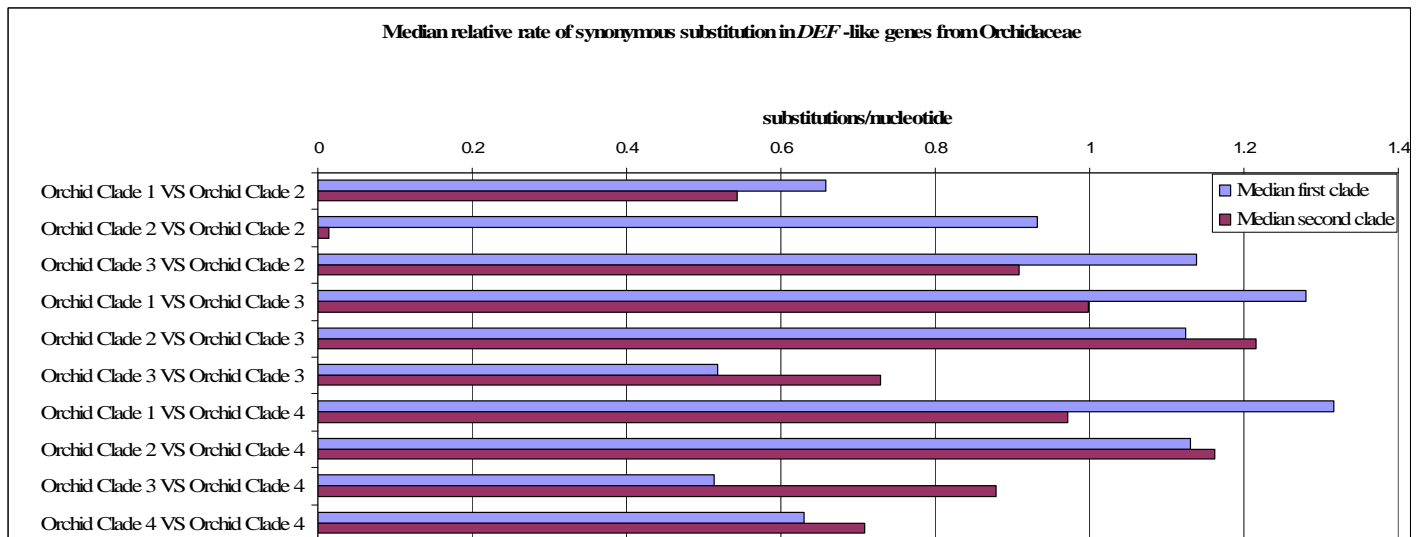

Supplement: Additional file 8 — Relative rates of A) nonsynonymous and B) synonymous substitution in DEF-like sequences from the Orchidaceae. Each bar represents the median of the corresponding relative rate of substitution between the pairs of two groups of sequences that yielded statistically significant results after correction for multiple comparisons. All data represented here are the result of two or more comparisons and exclusively involves orchid genes. [file 1471-2148-9-81-S8.pdf]
