# Supplementary material for: Positive selection and ancient duplications in the evolution of class B floral homeotic genes of orchids and grasses
Source: BMC Evol Biol. 2009 Apr 21;9:81. doi: 10.1186/1471-2148-9-81 (PMC2680841; doi:10.1186/1471-2148-9-81)
Supplement: Additional file 2 — Condensed alignment of DEF-like proteins from Asparagales included in the phylogenetic analysis illustrated by Figure 1 and Additional files 4 and 5. This alignment only includes variable amino-acid positions shaded according to their chemical property: Red background/green fonts = D, E; Red background/white fonts = H, K, R; Yellow background/green fonts = N, Q; Yellow background/black fonts = S, T; Bright green background/red fonts = L, I, V; Bright green background/white fonts = F, Y, W; Dark green background/red fonts = A, G; Dark green background/white fonts = M, C; White background/black fonts = P. [file 1471-2148-9-81-S2.pdf]

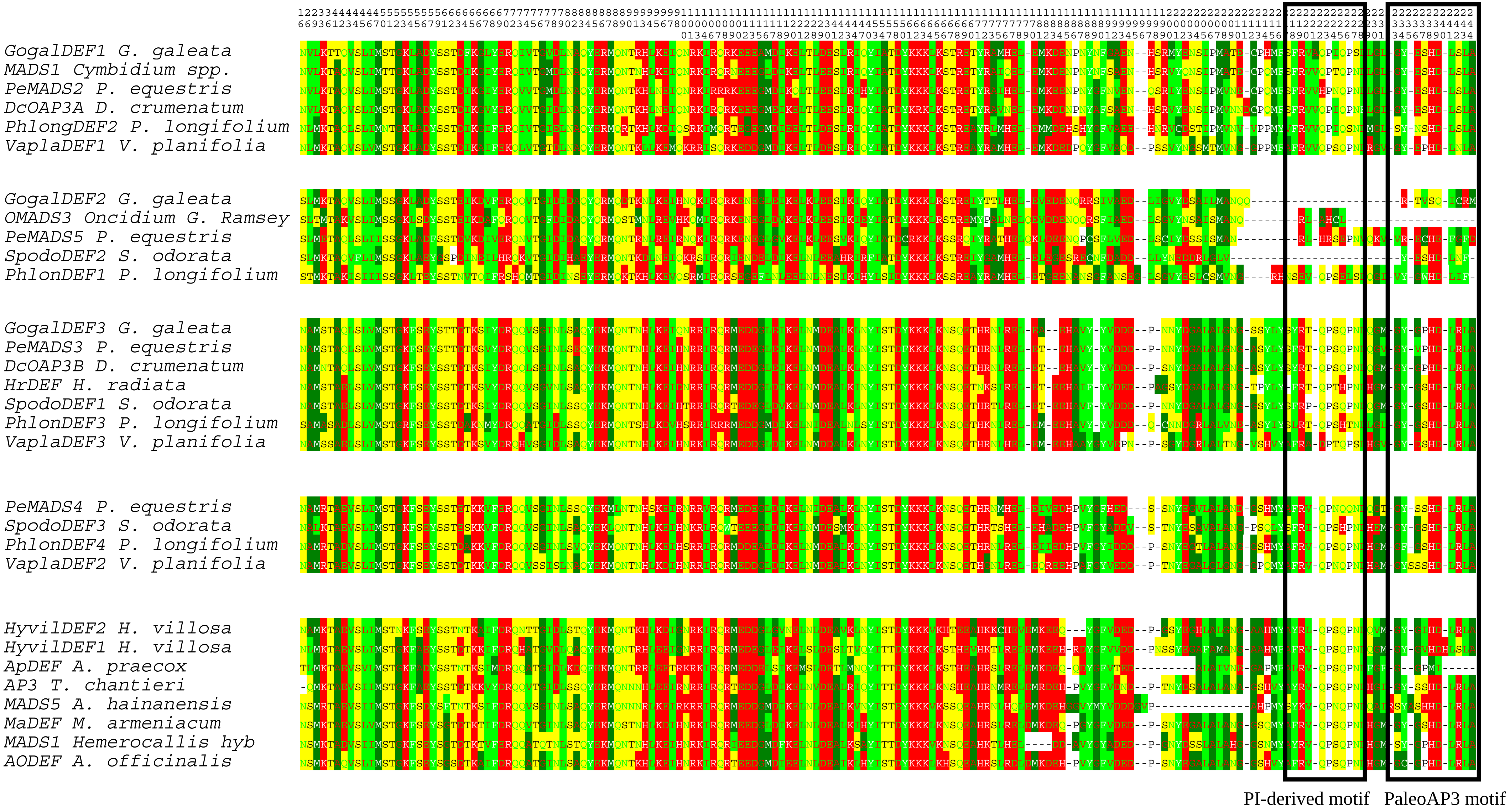
GogalDEF1 G. galeata
MADS1 Cymbidium spp.
PeMADS2 P. equestris
DcOAP3A D. crumenatum
PhlongDEF2 P. longifolium
VaplaDEF1 V. planifolia
GogalDEF2 G. galeata
OMADS3 Oncidium G. Ramsey
PeMADS5 P. equestris
SpodoDEF2 S. odorata
PhlonDEF1 P. longifolium
GogalDEF3 G. galeata
PeMADS3 P. equestris
DcOAP3B D. crumenatum
HrDEF H. radiata
SpodoDEF1 S. odorata
PhlonDEF3 P. longifolium
VaplaDEF3 V. planifolia
PeMADS4 P. equestris
SpodoDEF3 S. odorata
PhlonDEF4 P. longifolium
VaplaDEF2 V. planifolia
HyvilDEF2 H. villosa
HyvilDEF1 H. villosa
ApDEF A. praecox
AP3 T. chantieri
MADS5 A. hainanensis
MaDEF M. armeniacum
MADS1 Hemerocallis hyb
AODEF A. officinalis
PI-derived motif
PaleoAP3 motif
